# MIF/CXCR4 signaling axis contributes to survival, invasion, and drug resistance of metastatic neuroblastoma cells in the bone marrow microenvironment

**DOI:** 10.1186/s12885-022-09725-8

**Published:** 2022-06-17

**Authors:** Laura Garcia-Gerique, Marta García, Alícia Garrido-Garcia, Soledad Gómez-González, Montserrat Torrebadell, Estela Prada, Guillem Pascual-Pasto, Oscar Muñoz, Sara Perez-Jaume, Isadora Lemos, Noelia Salvador, Monica Vila-Ubach, Ana Doncel-Requena, Mariona Suñol, Angel M. Carcaboso, Jaume Mora, Cinzia Lavarino

**Affiliations:** 1 Developmental Tumor Biology Laboratory, Institut de Recerca Sant Joan de Déu, Barcelona, Spain; 2 Laboratory of Molecular Oncology, Pediatric Cancer Center Barcelona (PCCB), Hospital Sant Joan de Déu, Barcelona, Spain; 3 Department of Pathology, Hospital Sant Joan de Déu, Barcelona, Spain

**Keywords:** Neuroblastoma, Bone marrow, MIF, CXCR4, Hypoxia, 4-IPP

## Abstract

**Background:**

The bone marrow (BM) is the most common site of dissemination in patients with aggressive, metastatic neuroblastoma (NB). However, the molecular mechanisms underlying the aggressive behavior of NB cells in the BM niche are still greatly unknown. In the present study, we explored biological mechanisms that play a critical role in NB cell survival and progression in the BM and investigated potential therapeutic targets.

**Methods:**

Patient-derived bone marrow (BM) primary cultures were generated using fresh BM aspirates obtained from NB patients. NB cell lines were cultured in the presence of BM conditioned media containing cell-secreted factors, and under low oxygen levels (1% O_2_) to mimic specific features of the BM microenvironment of high-risk NB patients. The BM niche was explored using cytokine profiling assays, cell migration-invasion and viability assays, flow cytometry and analysis of RNA-sequencing data. Selective pharmacological inhibition of factors identified as potential mediators of NB progression within the BM niche was performed in vitro and in vivo.

**Results:**

We identified macrophage migration inhibitory factor (MIF) as a key inflammatory cytokine involved in BM infiltration. Cytokine profiling and RNA-sequencing data analysis revealed NB cells as the main source of MIF in the BM, suggesting a potential role of MIF in tumor invasion. Exposure of NB cells to BM-conditions increased NB cell-surface expression of the MIF receptor CXCR4, which was associated with increased cell viability, enhanced migration-invasion, and activation of PI3K/AKT and MAPK/ERK signaling pathways. Moreover, subcutaneous co-injection of NB and BM cells enhanced tumor engraftment in mice. MIF inhibition with 4-IPP impaired in vitro NB aggressiveness, and improved drug response while delayed NB growth, improving survival of the NB xenograft model.

**Conclusions:**

Our findings suggest that BM infiltration by NB cells may be mediated, in part, by MIF-CXCR4 signaling. We demonstrate the antitumor efficacy of MIF targeting in vitro and in vivo that could represent a novel therapeutic target for patients with disseminated high-risk NB.

**Supplementary Information:**

The online version contains supplementary material available at 10.1186/s12885-022-09725-8.

## Background

Neuroblastoma (NB) is the most common extracranial solid tumor diagnosed in the first 5 years of life and accounts for approximately 15% of all pediatric cancer-related deaths in patients younger than 15 years [1]. At the moment of diagnosis, half of NB patients present disseminated disease to sites such as bone marrow (BM), cortical bone, liver, and lymph nodes [2]. Among these, the BM is the most common site of dissemination and its infiltration at diagnosis or relapse occurs in more than 90% of stage 4 NBs [International Neuroblastoma Staging System (INSS)] [3]. The persistence of infiltrated BM during treatment is predictive of unfavorable outcome in patients [4, 5].

The BM microenvironment contains diverse cell types (hematopoietic and non-hematopoietic cells), as well as multiple cell-derived secreted factors and variable physical features that ensure the maintenance of normal hematopoiesis throughout the lifetime [6]. Mesenchymal stromal cells (MSCs), present in the BM stroma, play a pivotal role in the preservation of the Hematopoietic Stem Cell (HSC) niche through the production and release of cytokines and chemokines (i.e., SCF and CXCL12) [7]. HSC niches are also maintained by additional factors such as different oxygen tension and nutrient levels [8*,* 9]. BM is a highly vascularized tissue with an extensive arteriole and venous sinusoid branching network. This vasculature, together with the metabolic requirements of the cellular population, generates niches with different oxygen tension levels. Particularly, the HSC niches harbor low oxygen levels with local tension values as low as 1.3% [10].

This complex microenvironment is critical for the survival and invasion of NB tumor cells to the BM [11–13]. Recent studies have shown that the BM microenvironment promotes tumor cell migration, proliferation, and growth, and can contribute to drug resistance [14*,* 15]. However, little is known about the mechanisms involved in the tumor-promoting interaction that underlies the aggressive behavior of NB cells in the BM niche.

C-X-C Motif Chemokine Receptor 4 (CXCR4), a transmembrane receptor highly expressed on the HSC surface, plays a pivotal role in the development and establishment of the hematopoietic system in the BM. CXCR4 retains the HSC in their niches in response to the chemokine CXCL12 secreted by BM stromal cells [7]. In cancer, CXCR4 overexpression contributes to tumor growth, metastatic dissemination, and relapse [16]. In NB, overexpression of CXCR4 has been associated with malignant behavior of high-risk tumors [17]; however, its oncogenic role in relation with one of its ligands, CXCL12, has shown contradictory results [18]. CXCR4 is also a functional receptor for the cytokine Macrophage Migratory Inhibitor Factor (MIF), a pro-inflammatory cytokine [19]. Despite MIF is known to be secreted by NB cells [20*,* 21], the role of the MIF/CXCR4 axis in the maintenance and expansion of NB cells in the BM niche has never been studied.

In the present study, we aimed to identify molecular mechanisms that play a critical role in NB cell survival and progression in the BM niche. To accomplish our goal, we generated in vitro cell culture conditions that recapitulated critical features such as cytokine signaling, and low oxygen levels found in the BM microenvironment of metastatic NB patients. To this end, we combined conditioned media (CMs) obtained from patient-derived BM cell cultures with intrinsic low oxygen levels and NB cells. We explored in vitro and in vivo the effects of our BM conditions on NB cell proliferation, migration, invasion, and drug resistance. Our findings suggested that the tumor-promoting interaction between the BM microenvironment and NB cells is mediated, in part, by the MIF/CXCR4 signaling axis. Treatment with MIF inhibitor 4-Iodo-6-phenylpyrimidine (4-IPP) impaired the survival-promoting role of the BM environment, supporting MIF as an important mediator of NB aggressiveness in this niche. Altogether, our findings provide a new understanding of the contribution of the BM microenvironment to NB progression and reveal the antitumor activity of MIF inhibition. MIF could thus represent a novel therapeutic target that warrants further studies for more tailored NB treatment strategies.

## Methods

### Neuroblastoma and bone marrow cell cultures

Neuroblastoma (NB) cells LAN-1 (*MYCN* amplified, ALK F1174L, origin BM), SH-SY5Y (*MYCN* non-amplified, *ALK* F1174L, origin BM), IMR5 (*MYCN* amplified, *ALK* WT, origin abdominal mass), and the patient-derived cell line HSJD-NB01 (stage 4, *MYCN* amplified, origin suprarenal mass) were obtained from the repository of the Laboratory of Developmental Tumors, Hospital Sant Joan de Déu (HSJD), Barcelona and previously characterized in [22]. Cell cultures were maintained in regular culture conditions: RPMI-1640 supplemented with 10% of inactivated Fetal Bovine Serum (iFBS), 2 mM L-glutamine, and Penicillin/Streptomycin (100 μg/mL and 100 μg/mL, respectively) (Thermo Fisher Scientific, USA) in a controlled atmosphere at 37 °C and 5% CO_2_. Cell culture experiments were performed either at atmospheric oxygen levels [normoxia (Nx); 21% O_2_] or [hypoxia (Hx); 1% O_2_]. Oxygen levels were controlled with a modular incubator chamber MIC-101 (Billups-Rothenberg, Inc., USA) and cobalt chloride (CoCl_2_, Sigma-Aldrich, USA) was used as hypoxia-state control.

To establish patient-derived BM primary cultures, fresh BM aspirates were obtained from 20 NB patients treated in our institution. Samples were obtained at relapse or during consolidation therapy for NB (Additional Table 1). Samples were obtained under an Institutional Review Board approved protocol at Hospital Sant Joan de Déu, Barcelona, Spain. Parents and/or guardians signed informed consent before the collection of samples.

BM aspirates were collected and centrifuged in a density gradient Histopaque®-1077 (Sigma-Aldrich) at 2000 g for 20 minutes to obtain BM mononucleated cells. Cells were then plated and expanded in regular culture conditions up to 15 days before conditioned media generation. NB minimal residual disease (MRD) status within BM aspirates was detected by analysis of Paired-like Homeobox 2B gene (*PHOX2B*) gene expression using Real-Time Quantitative PCR, following previously described protocols [23] (Additional Table 1).

### Conditioned media (CM)

To generate CMs, we used in vitro expanded BM cells (2 × 10^6^; CM BM), BM cells co-cultured with NB cells in a ratio of 1:1 (1 × 10^6^ each; CM-BM/NB) or NB cells cultured alone (2 × 10^6^; CM-NB). After 48 hours in culture, CMs were collected, centrifuged at 1000 g for 10 minutes and kept at − 80 °C for future assays. Before each experiment, CMs were thawed and diluted 1:1 with regular RPMI without iFBS. As a control condition, CM-CNT was composed of complete RPMI-1640 with 5% of iFBS.

### Genomic data

Two publicly available RNA sequencing (RNA-seq) data sets were used for the study. GSE94035 comprised RNA-seq data from 16 primary tumors, 42 BM-derived disseminated NB tumor cells and corresponding 28 BM-derived non-tumor cells of NB patients [12], and GSE62564 comprised 498 primary NB diagnostic biopsies, including INSS (International Neuroblastoma Staging System) stage 1-3 and 4S as well as stage 4 NB specimens. In addition, GSE62564 data set were analyzed according to the classification criteria of High-Risk NB Study 1.7 of SIOPEurope (SIOPEN) (NCT01704716). Nine samples were not included in the study due to a lack of available clinical information [24]. The Contal-O’Quigley method was used to establish the threshold for overall survival (OS) studies (Kaplan-Meier method) [25]. The following thresholds were obtained: *MIF* (7.1; log-rank *p < 0.0001*), *CXCL12* (3.5; log-rank *p < 0.0001*), *CXCR4* (5.9; log-rank *p = 0.00018*), *CD74* (7.4; log-rank *p < 0.0001*), *CD44* (7.7; log-rank *p < 0.0001*), *CXCR7* (2.9; log-rank *p = 0.59*), and *CXCR2* (0.8; log-rank *p = 0.018*). The log-rank test was used to analyze survival differences performed with GraphPad Prism 8 (La Jolla, USA).

### MIF and CXCR4 inhibitors

AMD-3100 (Plerixafor), ISO-1 (Selleckchem, Germany), and 4-Iodo-6-phenylpyrimidine (4-IPP) (Merck, Germany) were diluted with ethanol at a stock concentration of 10 mM. Cell viability was assessed at different concentration ranges using MTS assay (Promega Inc., USA). NB cell lines and fresh BM-MNCs were cultured in 96-well plates for 24 hours in regular media before being treated with drugs at concentration ranges: AMD-3100 (50-0.0001 μM), ISO-1 (50-0.2 μM), 4-IPP (50-0.2 μM) (*n =* 6 per each concentration). After 72 hours, cell viability was compared to control wells (vehicle 100% viable). The half-maximal inhibitory concentration (IC50) of tumor cell viability was calculated with a nonlinear regression log (inhibition) vs. response variable slope using the software Prism 8 (GraphPad, USA).

### Cell viability in bone marrow-based in vitro conditions

To test CM-driven changes in NB cell viability, LAN-1 and SH-SY5Y (2 × 10^3^ cells per well), HSJD-NB01 (8 × 10^3^ cells per well) and IMR5 (8 × 10^3^ cells per well) cell lines were plated in 96-well plates with 100 μL of regular media for 24 h. Regular culture media was then replaced by CM-NB, CM-BM, CM-BM/NB, CM-CNT and cultured under normoxic or hypoxic conditions (*n* = 6 replicates per condition). Cell viability was measured with MTS assay (Promega) at 0, 24, 48, 72 and, 96 h at 490 nm with microplate reader Infinite® 200 PRO (Tecan, Switzerland). Cell viability was represented as a relative fold-change compared to the initial MTS signal at time 0 h. To assess whether treatments could affect NB cell viability in the presence of CMs, the same protocol was performed adding 5 μM of 4-IPP, 10 nM of AMD3100, 10 ng/mL recombinant human MIF (SRP3321, Millipore Sigma) or vehicle.

### siRNA mediated knockdown

Human *CXCR4* gene was transiently silenced in LAN-1 and SH-SY5Y neuroblastoma cells using Dharmacon ON-Targetplus Human CXCR4 siRNA technology (Horizon Discovery, USA). Cells were transfected with Lipofectamine® RNAiMAx transfection Reagent (Invitrogen, USA) and a 10 nM mixture of SMARTpool siRNA against human *CXCR4* or a ON-TARGETplus Non-targeting Control Pool, following manufacturer’s indications.

### Cell migration and chemoattraction assays

NB cells (8 × 10^5^ cells per well) were plated into 6-well plates and allowed to reach a confluent monolayer. The monolayer was scratched with a pipette tip, and regular cell culture media was replaced by CM and incubated in normoxic or hypoxic conditions for 24 h. Cells were also treated with 5 μM of 4-IPP or 10 nM of AMD3100 in CM-BM/NB. After 24 h, the percentage of the cell-free area was measured with ImageJ Plugin Wound Healing Tool by RIO Imaging. Cell chemoattraction and invasion were tested using 8.0 μM pore membrane Cell Culture Insert Transwells (Corning, USA) pre-coated with 10% of Matrigel (Corning, USA) in RPMI media. Transwell upper chambers were filled with 2 × 10^5^ cells per chamber in 200 μL of regular media with 1% iFBS, whereas lower chambers were filled with 500 μL of each CM and incubated in normoxic and hypoxic conditions for 96 h. Finally, invaded cells were fixed and stained with 4% paraformaldehyde for 10 min and 2% crystal violet for 30 min. In experiments using inhibitors drugs, cells were pre-treated overnight with 5 μM of 4-IPP or 10 nM of AMD3100 and then plated maintaining drug treatment in Transwell’s upper chambers. The surface area invaded by NB cells was measured in three representative areas per Transwell membrane using light microscopy (20x; DM 5000 B Leica, Germany) with ImageJ Software [26].

### Drug response studies

LAN-1 cells (2 × 10^3^ cells per well) were plated in 100 μL of CM-CNT or CM-BM/NB under normoxia or hypoxia for 24 h. Drugs were added at different concentration ranges: doxorubicin (2500-0.381 nM), etoposide (3750-0.572 nM), and SN-38 (500-0.076 nM). After 72 h, an MTS assay (Promega) was performed and IC50 concentrations were determined for each different drug and culture condition. To assess whether drug response could be modulated after MIF inhibition, the same protocol was performed adding to each experimental condition 5 μM of 4-IPP, or vehicle together with chemotherapeutic agents. Results are expressed as the percentage of relative viability, in which each experimental conditions were compared to their own control.

### RNA extraction and RT-qPCR

Total RNA was isolated using TRI Reagent (Sigma-Aldrich) and quantified with NanoDrop spectrophotometer (Thermo Fisher Scientific). cDNA was generated with M-MLV reverse transcriptase system (Thermo Fisher Scientific) using 1 μg of total RNA as previously reported [27]. Gene expression was quantified using SYBR® Green PCR Master Mix in a QuantStudio 6 Real-Time PCR system (Thermo Fisher Scientific). Sequences and references for primers are listed in Additional Table 2. Relative expression of mRNA was determined using the 2^-(ΔΔCt)^ quantification method and normalized to *YWHAZ* as a reference gene [28].

### Proteome arrays

Determination of cytokine-expression levels was performed using Proteome Profiler Human Cytokine Array Kits (R&D Systems, USA) following the manufacturer’s protocols. The chemiluminescent signal was detected with the iBright Imaging System (Thermo Fisher Scientific) and quantified with QuickSpots Software (R&D Systems).

### Enzyme-linked immunosorbent assay (ELISA)

CXCL12 and MIF secretion in CM was measured using the Human CXCL12/SDF-1 DuoSet ELISA and MIF Quantikine ELISA kits (R&D Systems) following the manufacturer’s protocols. The absorbance was measured at 450 nm and corrected at 540 nm on a microplate reader Infinite® 200 PRO (Tecan, Switzerland).

### Immunoblotting

Cell samples were lysed with RIPA 1X buffer (50 mM Tris pH 8.8, 150 mM NaCl, 0.1% SDS, 0.5% sodium deoxycholate and 1% NP40) plus complete Proteases Inhibitor Cocktail Tablets (Roche, Switzerland). Protein extracts were quantified with Bradford Reagent (BioRad, USA). Samples were separated by electrophoresis in polyacrylamide gels (8 - 12%), transferred to nitrocellulose membranes (Thermo Fisher Scientific) and PageRuler™ Plus Prestained Protein Ladder as protein size control (Thermo Fisher Scientific). Immunoblot detection was performed with near-infrared labeled secondary antibodies in the imaging system Odyssey® CLx (LI-COR Inc., USA) and software Image Studio® (LI-COR Inc., USA) following manufacturer’s instructions. Primary and secondary antibodies are listed in Additional Table 3. Relative protein levels were measured, and ratios calculated (phosphorylated/total) by ImageJ Software.

### Flow cytometry

Cell cultures were washed in blocking-PBS buffer [1% Bovine Serum Albumin (Sigma-Aldrich) and 1% FBS in PBS 1X (Thermo Fisher Scientific)], stained with primary-conjugated antibodies (Additional Table 3), as well as with 0.2 μg/mL 4′,6-Diamidine-2′-phenylindole dihydrochloride (DAPI; Sigma-Aldrich) to select viable cells. Flow cytometry data were analyzed with ACEA Novocyte 3000 (Acea Bioscience Inc., USA), and results were processed with NovoExpress Software (Acea Bioscience Inc.)

#### BM characterization

On day 15 of BM expansion, BM cultures (*n* = 6) were washed and stained with two panels of fluorochrome-conjugated antibodies (BD Bioscience, USA). A primary culture from non-oncologic BM-derived mesenchymal stem cells (BM-MSCs) was used as a control sample for mesenchymal markers. Panels: Panel 1 included CD11b-PE, CD105-FITC, CD45-V500, CD34-PerCP, CD19-PeCy7, and CD123-APC. Panel 2 included CD90-PE, CD45-V500, HLA-DR-PerCP, CD19-PeCy7, and CD117-APC. The percentages of mesenchymal cells were calculated as CD45^−^/CD90^+^/HLA-DR^+^ and CD45^−^/CD105^+^, whereas percentages for the rest of markers were defined as positive stained events over the CD45^+^ population. Sample acquisition was performed in a FACSCanto II (BD Bioscience, USA) and all channels were previously compensated for fluorescence spillover.

#### CXCR4 and CD74 expression

NB cells cultured with CM-CNT, CM-NB, CM-BM, CM-BM/NB, under normoxic or hypoxic conditions were collected with Versene (Thermo Fisher Scientific) at 72 h and exposed to primary-conjugated antibodies for 30 min at room temperature protected from light exposure. Basal cell surface expression of CXCR4 and CD74 was measured in LAN-1, SH-SY5Y and IMR-5 cells under regular culture conditions using the same method.

#### In vitro co-culture studies

Co-cultures combining NB and fresh BM-MNCs cells were performed to evaluate the specific vulnerability of tumor cell lines but not BM cells to MIF inhibition. Thus, NB cells were plated in 6-well plates (5 × 10^3^ cells *per* well) for 24 h in regular media. Then, BM cells were added in a ratio of 10:1 (BM:NB) together with 30 μM of 4-IPP or vehicle, in regular media. NB and BM cells cultured alone were used as positive and negative controls, respectively. After 72 h, floating and attached cells were collected, washed, and stained with primary-conjugated antibody for 30 min at room temperature avoiding light exposure.

### In vivo assays

Animal procedures were performed at the animal facility at HSJD according to regulatory order (214/97, Generalitat de Catalunya) and the local animal ethics committee (9330 CEEA, Universitat de Barcelona). Research with mice adhered to European regulations and ARRIVE guidelines and approved by the animal experimental Ethics Committee of the *Universtidad de Barcelona* and the *Generalitat de Catalunya* (9330 CEEA; animal protocol number 214/97).

Xenograft models were generated in 5-6 weeks old female athymic nude mice (Envigo, Spain). LAN-1 cells were injected subcutaneously (*s.c*.) at both flanks in a mixture of Matrigel® and RPMI medium (vol/vol). Every 3-4 days animals were followed up by measuring weight and tumor size with a caliper, as well as monitored for macroscopic metastasis events. Tumor size (mm^3^) was calculated under the formula: 1/2(length × width^2^) [29]. Endpoint criteria were set when tumor size reached ≥1500 mm^3^ at a single flank or animals lost 20% of the weight. Engraftment and survival analyses were calculated by Kaplan-Meier Method performed with Prism 8 software (GraphPad, USA).

#### Tumor growth under stromal support

LAN-1 cells were injected *s.c.* alone (5 × 10^5^ cells; NB group; *n* = 18) or together with BM expanded cells in a 1:1 ratio (5 × 10^5^ each cell type; BM/NB group; *n* = 22) to compare tumor engraftment (tumor size ≥100 mm^3^), progression, and survival. As a negative control, a small group of mice was injected with BM cells alone (group BM*, n* = 4). Data gathered from two different in vivo experiments are represented together. Tumor masses were harvested and fixed in 4% formaldehyde solution for posterior immunohistochemistry (IHC) studies.

#### In vivo treatment

Antitumor efficacy of the MIF inhibitor 4-IPP (Merck) was tested in LAN-1 xenografts. For in vivo treatment, 4-IPP was diluted in corn oil (Sigma-Aldrich) as previously described [30]. LAN-1 cells (1.5 × 10^6^ cells) were injected *s.c.* in both flanks and 7 days later mice were randomly distributed (vehicle, *n* = 14) and treatment (4-IPP, *n* = 10) groups. 4-IPP treatment was administrated via intraperitoneal route 3 days a week every 2 days for 4 weeks at 80 mg/kg.

### Immunohistochemistry

After fixation, tissues were embedded in paraffin by conventional automated systems. Four-micron sections were stained with hematoxylin-eosin (H/E), human nuclei antibody, and Ki-67 (Additional Table 3).

### Statistical analyses

Unless otherwise specified, group differences were calculated comparing each experimental condition to control (CM-CNT under normoxia) with Two-way ANOVA and Dunnett’s multiple comparison test. Survival curves were estimated by Kaplan-Meier method and compared among groups by log-rank test. Statistical analysis was performed with the software Prism 8 (Graphpad, USA). Significance was defined as *P < 0.05* or *P < 0.01*.

## Results

### Characterization of cell-secreted factors in the bone marrow microenvironment of high-risk neuroblastoma

**Fig. 1:**
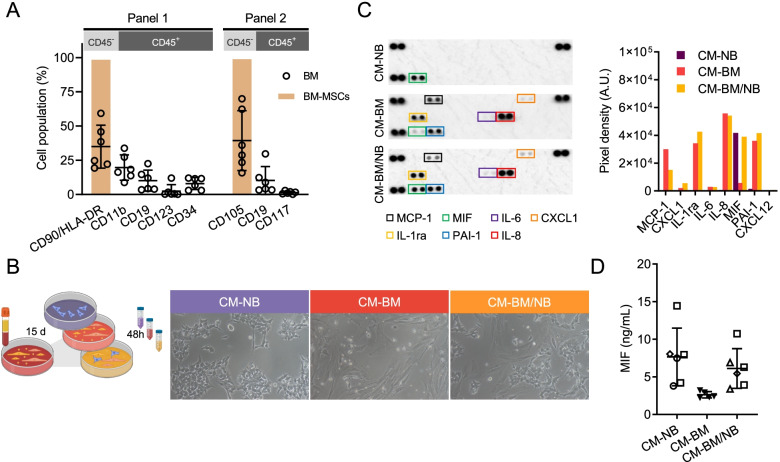
Generation and characterization of CMs mimicking the BM niche*.* **A)** Quantification of different cell populations in expanded BM cultures (*n* = 6) by flow cytometry (empty dot plots). Purified culture of BM-MSC (column bars) was used as positive control for stroma markers (CD90, CD105 and HLA-DR). **B)** Graphical scheme of CM’s production (left). In vitro image (10x objective) of cells used for CM’s production at 48 h (right). **C)** Cytokine Profile Array membranes of CM-NB, CM-BM, CM-BM/NB (left). Quantification of detected cytokines and chemokines represented as mean pixel density of duplicate spots (right). **D)** Extracellular MIF quantification by ELISA in CM-NB (*n =* 6), CM-BM (*n* = 5) and CM-BM/NB (*n =* 6), being LAN-1 square, SH-SY5Y rhomboid, and IMR5 a circle

To mimic part of the complex microenvironment of the bone marrow (BM) in vitro, we recapitulated some features of this niche such as the cell-secreted factors produced by stroma enriched BM cultures. We established BM cultures from mononucleated cells (MNC), using 20 neuroblastoma (NB) patient-derived primary BM aspirates obtained from 14 high-risk (HR) NB patients at relapse or during consolidation treatment. Relative expression of the minimal residual disease PCR target *PHOX2B* was undetectable (Additional Table 1). After 15 days, the BM cell cultures displayed a heterogeneous population of adherent cells with diverse phenotypes as assessed by phase-contrast microscopic analysis. To characterize the different cell populations, BM cultures were stained with lineage-specific cell surface markers and analyzed by flow cytometry (BM cultures *n* = 6, each one from a different patient). A predominant cell population was positive for mesenchymal stem cell (MSC) surface markers, including CD90/HLA-DR and CD105 (34.94 ± 15.71% and 39.33 ± 21.86%, mean ± SD, respectively). BM MSC derived from a BM of a non-oncologic patient were used as a positive control for mesenchymal cell markers. BM cultures also stained for the myeloid-linage marker CD11b (19.66 ± 9.21%, mean ± SD), and lymphoid-lineage marker CD19 (10.66 ± 7.6%, mean ± SD). Minor cell populations stained for dendritic cell marker CD123 (2.46 ± 4.69%, mean ± SD), hematopoietic cell markers CD34, and CD117 (7.96 ± 4.32% and 1.34 ± 1.02%, mean ± SD, respectively) (Fig. 1A).

After 15 days of expansion, BM cells were cultured alone or in the presence of NB cells (LAN-1, SH-SY5Y, IMR5 and HSJD-NB01) to resemble the tumor-modified BM niche. After 48 h, supernatants were collected to obtain the cell-secreted factors in the form of conditioned media (CM). With this approach, we generated cell culture media from BM primary cells cultured alone (CM-BM) or co-cultured with NB cells (CM-BM/NB), and NB cells cultured alone (CM-NB) (Fig. 1B). All CMs were characterized using a cytokine profiling array. Seven cytokines were detected in both CM-BM and CM-BM/NB; MCP-1, CXCL1, IL-1ra, IL-6, IL-8, MIF, and PAI-1 (Fig. 1C). Interestingly, MIF was the only cytokine identified in the CM-NB, and at similar levels in CM-BM/NB. The high concentrations of MIF protein in CM-NB and CM-BM/NB were confirmed by ELISA assay (concentrations of 7.65 ± 3.83, 2.62 ± 0.42, and 6.16 ± 2.64 ng/mL in CM-NB, CM-BM, and CM-BM/NB, respectively) (Fig. 1D). CXCL12 was not detectable in the CMs, being below the detection limit in all the studied conditions (data not shown). These findings suggested that MIF was secreted by NB cell lines when cultured alone or in the presence of BM cells. Altogether, these results highlighted MIF as a relevant cytokine potentially involved in the interaction between NB cells and the BM-niche.

### MIF and CXCR4 expression are associated with disseminated high-risk neuroblastoma and poor prognosis

**Fig. 2:**
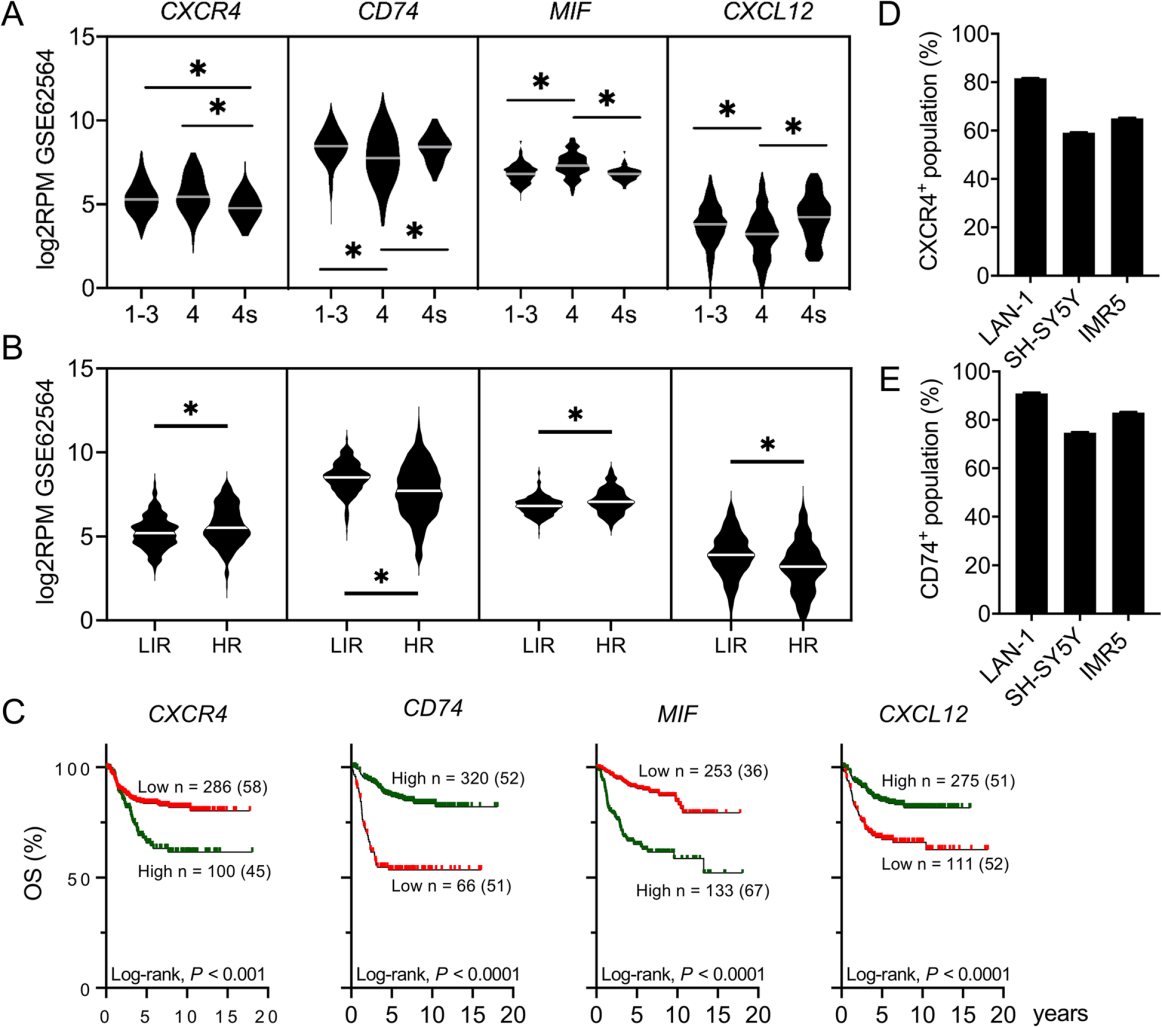
*MIF* and *CXCR4* are expressed in high-risk NB tumors and cell lines. Violin plots showing *CXCR4*, *CD74*, *MIF* and *CXCL12* gene expression across **A)** different NB stages and **B)** risk groups, low/intermediate-risk (LIR) and High-risk (HR) (GSE62564). *P < 0.01 (One-way ANOVA, Kruskal Wallis test). **C)** NB patient overall survival associated with *CXCR4*, *CXCL12*, *CD74* and *MIF* expression levels (GSE62564, Kaplan-Meier analysis). High expression is shown in green and low expression in red. Total number of patients and (positive events) *per* group are also shown. Percentage of **D)** *CXCR4* and **E)** *CD7*4 positive cells in a NB cell line panel analyzed by flow cytometry (*n* = 3)

Increased expression of *MIF* has been reported in diverse cancers, including NB. Here, we explored the MIF/CXCR4 pathway in primary NB tumor biopsies and BM-derived disseminated NB tumor cells (DTCs) using publicly available gene expression datasets. Expression profiles of *MIF*, *CXCL12*, and the receptors *CXCR4*, *CXCR2*, *CXCR7*, *CD74*, and *CD44* were first analyzed using an RNA sequencing (RNA-seq) dataset (GSE62564) of 498 primary NB tumor samples (Additional Fig. 1A) [24]. All genes were found to be expressed across NB clinical risk groups. Expression of *MIF* was highest in stage 4 (clinically high-risk disseminated) tumors as compared to stage 4S (low-risk disseminated) tumors and non-disseminated tumors (stage 1-3). *CXCR4* showed lower expression in stage 4S tumors than stage 4 and non-disseminated tumors (Fig. 2A). Stage 4S tumors also showed lower expression of *CXCR4* than stage 4 and non-disseminated tumors. In contrast, *CD74* and *CXCL12* showed lower expression in stage 4 NB (Fig. 2A). The same dataset was analyzed according to the high-risk Neuroblastoma Study 1.7 of SIOPEurope (SIOPEN) stratification risk, where HR-NB is defined as stages 2, 3, 4, and 4S with *MYCN*-amplification and stage 4 *MYCN* non-amplified > 12 months at diagnosis. The remaining patients are considered as low/intermediate-risk (LIR). Higher *CXCR4* and *MIF*, but lower *CD74* and *CXCL12* gene expression, was observed for HR patients when compared to LIR patients (Fig. 2B). Accordingly, expression was associated with overall survival of NB patients, being a high expression of *CXCR4* or *MIF* associated with poor overall survival of patients (*P < 0.01*, both), whereas a more favorable outcome was related to high expression of *CD74*, *CXCL12,* and *CD44* (*P < 0.01* each of them) (Fig. 2C and Additional Fig. 1B).

Next, we analyzed the publicly available RNA-seq dataset GSE94035 including 16 primary HR-NB tumors and 42 samples of BM-derived NB DTC (22 at diagnosis and 20 at relapse of disease) (Additional Fig. 1C) [12]. The analysis revealed a positive correlation of *MIF* transcript levels with the percentage of BM DTC enrichment. Additionally, DTC enrichment levels positively correlated with BM expression levels of *PHOX2B*, a specific NB minimal residual disease (MRD) marker [23]. In contrast, the expression of *CXCR4*, *CD74*, *CXCR7*, *CD44*, and *CXCR2* showed an inverse correlation with the percentage of DTC-enrichment. These results could be due to the high expression of these receptors in the BM by MNCs. A similar correlation was observed with the expression of *PTPRC* (*CD45*), a marker for hematopoietic cells absent in NB cells (Additional Fig. 1C) [31]. No differences in MIF expression were observed between primary tumor samples and DTC samples at diagnosis (data not shown). The presence of MIF receptors –CXCR4 and CD74– in the cellular membrane of our NB cell lines panel was confirmed by flow cytometry (Fig. 2D-E and Additional Fig. 1D). These findings showed that increased expression of *CXCR4* and *MIF* is associated with more aggressive, disseminated disease, and suggested NB cellularity as the main source of MIF within disseminated BM samples.

### Bone marrow environment promotes neuroblastoma viability, invasion, and drug resistance in vitro

In addition to cytokine signaling, the BM microenvironment is characterized by low oxygen tension, particularly in the hematopoietic stem cell niches [32]. To mimic these hypoxic niches, we cultured NB cells with CMs (CM-NB, CM-BM, CM-BM/NB or CM-CNT) under different oxygen levels; high (atmospheric oxygen 21%; herein named normoxia) or low (physiological BM 1% O_2_, 5% CO_2_ and 94% N_2_; herein hypoxia) oxygen levels. The hypoxic state of cell culture was maintained in a modular chamber, and validated by using cobalt chloride (CoCl_2_), a hypoxia-chemical mimetic. Hypoxic conditions were confirmed by analyzing HIF-1α protein levels, and the expression of *HIF-1α* and target genes (Additional Fig. 2A-B) [8].

**Fig. 3:**
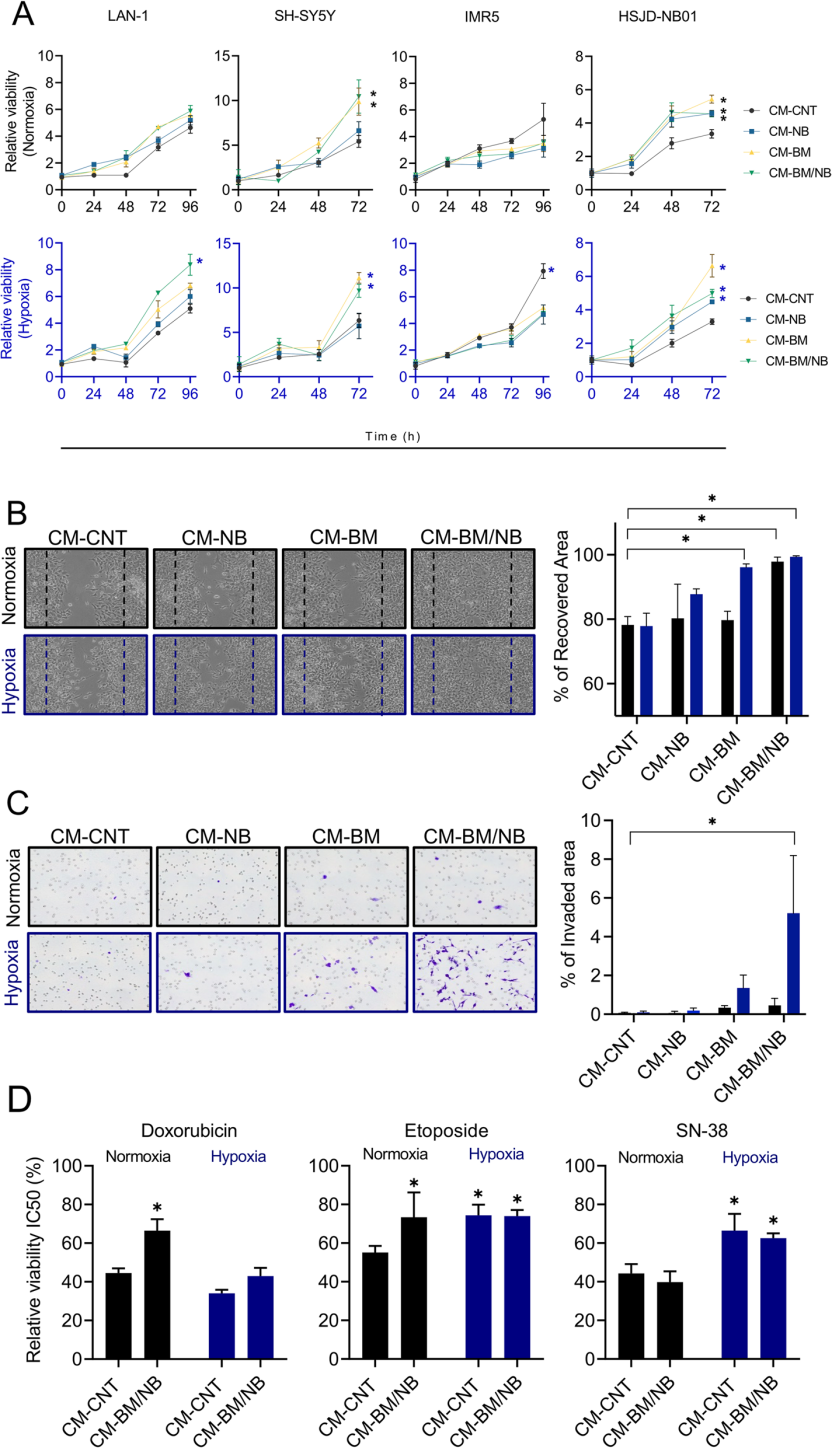
BM-like culture conditions promote NB proliferation, invasion, and drug resistance. **A)** LAN-1, SH-SY5Y, IMR5, and HSJD-NB01 cells were cultured in CMs under normoxic, and hypoxic conditions and MTS assay was performed over 4 days. Data are represented as a fold change viability to time 0 h (*n* = 6). **B)** Wound healing assay of LAN-1 cells after 24 h (left) and its quantification (right). Differences were calculated comparing each experimental condition to control (CM-CNT in normoxia) (*n =* 3). Normoxia conditions (black) hypoxia conditions (blue). Two-way Anova, Dunnett’s multiple comparison test. **C)** Transwell invasion assay of LAN-1 cells after 96 h (left) and its quantification (right). Differences were calculated comparing each experimental condition to control (CM-CNT in normoxia) (*n* = 3). Two-way Anova, Dunnett’s multiple comparison test. **D)** Activity of chemotherapy (doxorubicin, etoposide, and SN-38) over LAN-1 cells cultured with CM-BM/NB at 72 h (*n =* 3). Normoxia conditions (black) hypoxia conditions (blue). (*) *P <* 0.05

We studied the effect of our BM-like experimental conditions on NB cell viability, invasion, chemoattraction, and drug response. Cell viability showed a time-dependent increase in LAN-1 and SH-SY5Y cell lines and in the patient-derived HSJD-NB01 when cultured under the in vitro BM-like conditions (i.e., CM-BM and CM-BM/NB condition), as compared to controls (Fig. 3A). In LAN-1 cells, this effect was enhanced under hypoxic conditions, whereas oxygen levels did not affect SH-SY5Y and HSJD-NB01 cell viability. In contrast, exposure to hypoxia increased significantly IMR5 cell viability, whereas no effect was observed for BM/NB CMs. The addition of recombinant MIF to regular medium conditions did not induce changes in cell proliferation (Additional Fig. 3A), even in cells with downregulated CXCR4 by siRNA (Additional Fig. 3B-C).

LAN-1 cells were subsequently used for functional studies due to their unique response to CMs and hypoxia compared to the other NB cell lines. In vitro wound-healing assays showed enhanced migration capacity of LAN-1 cells after 24 h of exposure to CM-BM and CM-BM/NB under hypoxic conditions (both, *P < 0.01*) (Fig. 3B). Similarly, in chemotaxis experiments, we observed a significant increase in the capacity of LAN-1 cells to invade the transwell matrix and migrate towards CM-BM/NB (percentage of the invaded area of 5.21 ± 2.97; mean and SD; *P < 0.01*, Fig. 3C). These findings proved that the CM-BM/NB had a clear chemo-attractant effect on LAN-1 cells, inducing cell migration and invasion across the 3-D Matrigel matrix barrier.

We thus explored the impact of CM-BM/NB on NB cell response to drugs. We tested the response to doxorubicin, etoposide, and SN-38, drugs included in current chemotherapy protocols for patients with HR-NB [33, 34]. Under control conditions (CM-CNT and normoxia), IC50 values were 27.43 nM (22.17-34.3 nM) for doxorubicin, 491.3 (447.4-546.8 nM) for etoposide and 1.49 nM (1.34-1.65 nM) SN-38. Our BM-based model affected LAN-1 sensitivity in a drug-specific manner. Doxorubicin showed higher IC50 values and corresponding less cytotoxicity in cells cultured with CM-BM/NB, being significant in normoxia conditions (*P < 0.05*), whereas etoposide showed increased IC50 values in all culture conditions as compared to CM-CNT at normoxia and SN-38 resistance was promoted by the hypoxic conditions (Fig. 3D). The high IC50 values observed for etoposide at basal levels suggested intrinsic LAN-1 resistance to this drug, independent of the BM cell culture conditions. Thus, CM-BM/NB increased LAN-1 resistance to drugs such as doxorubicin and etoposide.

Our findings suggested that cell-derived secreted factors in CM-BM/NB and hypoxic conditions potentially contributed to promoting tumor aggressive phenotypes like NB-cell proliferation, migration, and invasion.

### Bone marrow cells enhance engraftment in a neuroblastoma xenograft in vivo model

**Fig. 4:**
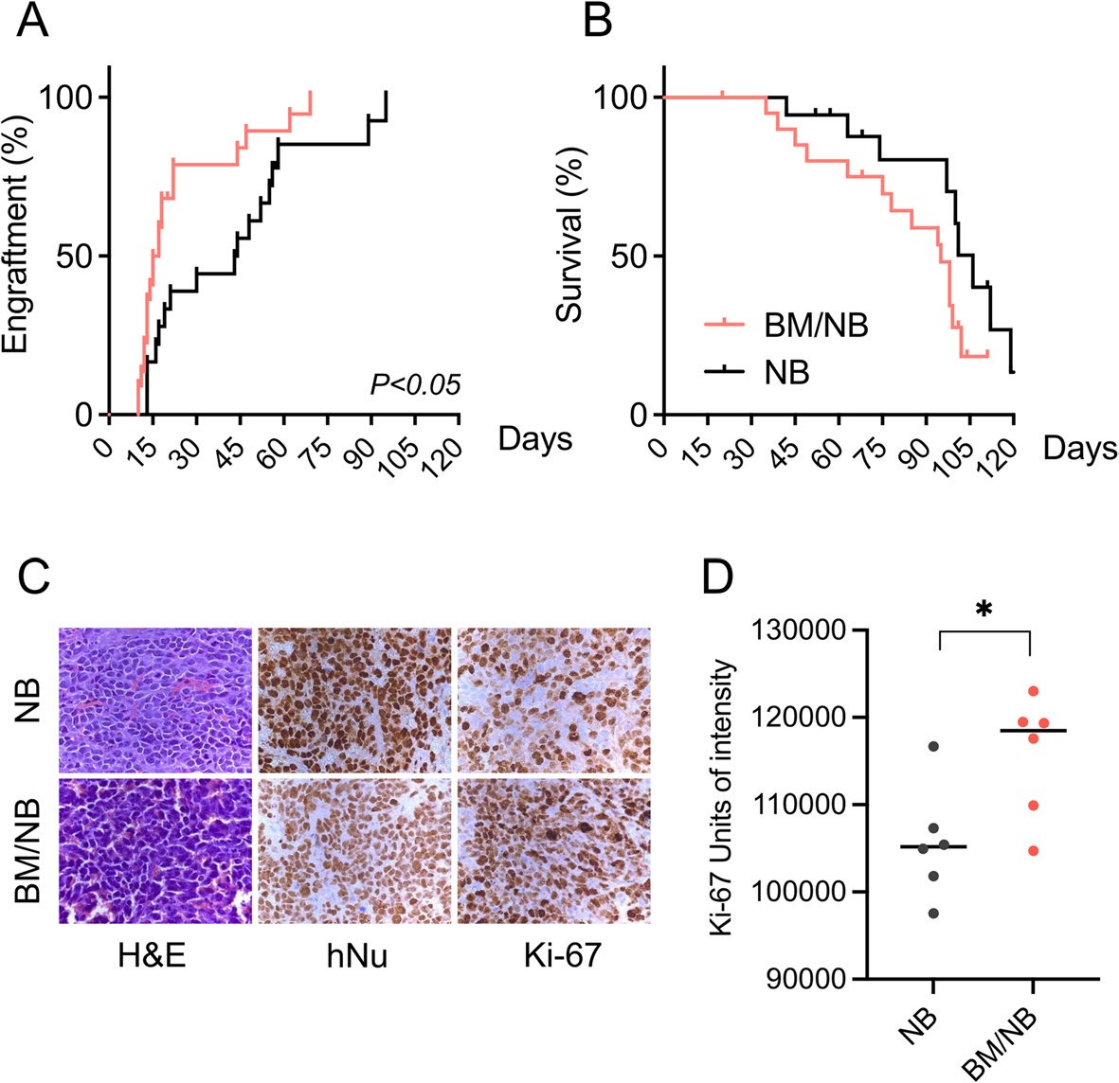
In vivo effects of BM in a LAN-1 xenograft model: **A)** Engraftment curves for each group, reported at 100 mm^3^ tumor size. **B)** Tumor survival curves for each group, reported at 1500 mm^3^ tumor size. **C)** Histology of primary subcutaneous LAN-1 tumors by H&E, hNu, and Ki-67 staining (40x). **D)** Ki-67 quantification of stained FFPE sections (*n =* 6) was quantified using ImageJ software. Mann-Whitney test. (*) *P < 0.05*

To further investigate the impact of BM cells on NB cell tumorigenicity, we injected subcutaneously into the flank of athymic nude mice LAN-1 cells (5 × 10^5^ per flank) either alone (NB group, *n* = 18) or mixed with expanded BM cells (1:1 ratio; BM/NB group, *n* = 22). As a negative control, a small group of mice was injected with BM cells alone (BM group, *n* = 4). Time to tumor cell engraftment, tumor growth rate, morphology, and recipient mice survival were assessed until tumors reached the end-point criteria. All tumors from NB and BM/NB groups achieved 100% of engraftment, however, the median engraftment time was significantly shorter in BM/NB as compared to the NB group (16 vs. 43.5 days; *P < 0.05*) (Fig. 4A).

As expected, no engraftment or evidence of disease was observed in the BM group (data not shown). The BM/NB group showed no significant differences in survival time when compared to the NB group (95 vs. 106 days; *P =* 0.1) (Fig. 4B). Immunohistochemical analysis of the proliferation marker Ki-67 in formalin-fixed paraffin-embedded (FFPE) tumor sections showed a significant increase of positive cells in tumors of the BM/NB group (Fig. 4C-D). These results showed that BM cells contributed to generate a favorable microenvironment that promoted NB cell engraftment and tumor growth.

### Bone marrow environment increases MIF receptor CXCR4 and activates intracellular signaling pathways

**Fig. 5:**
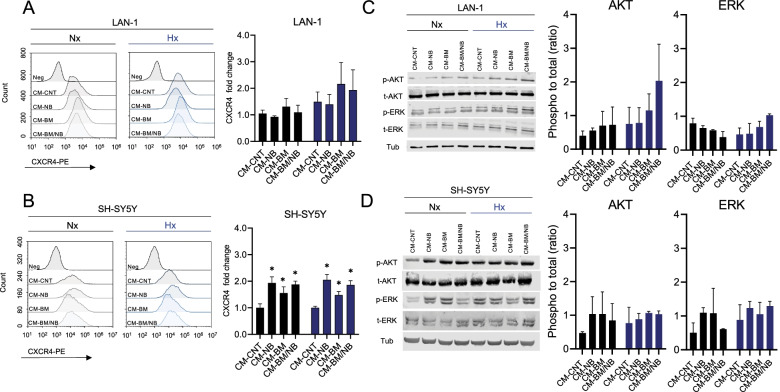
BM-like culture conditions increase CXCR4 surface expression and activate AKT and ERK pathways. **A)** Flow cytometry histograms of CXCR4 expression in LAN-1 cells cultured under BM-like conditions for 72 h (left). CXCR4 expression is represented as a fold-change to CM-CNT Nx (*n =* 3; right). **B)** Flow cytometry histograms of CXCR4 expression in SH-SY5Y cells cultured under BM-like conditions for 72 h (left). CXCR4 expression is represented as a fold-change to CM-CNT Nx (*n =* 3; right). Histograms were generated with NovoFlow Software (Acea Bioscience). Normoxia results are in black and hypoxia in blue. (*) *P < 0.01* (Two-way ANOVA, Dunnett’s multiple comparison test). Immunoblots of LAN-1 **C)** and SH-SY5Y **D)** cultured under BM-like conditions for 48 h. Band intensity p-AKT (S473), p-ERK (T202/Y204 T185/Y187) ratios are calculated by measuring the relative protein levels (phosphorylated/total), and γ-Tubulin (as a loading control) by using ImageJ Software (*n* = 2)

We next explored whether the BM-like conditions, in the form of CMs and hypoxia, affected the levels of the MIF receptors (CXCR4 and CD74). To assess this, we evaluated receptor levels on the NB cell surface by flow cytometry. LAN-1 showed no differences on cell surface expression of CXCR4 upon exposure to CM-BM and CM-BM/NB when compared to controls (fold change of 2.2 ± 0.8 and 1.9 ± 0.8 respectively). In SH-SY5Y, CXCR4 expression increased after exposure to all CM, both in hypoxic and normoxic conditions (Fig. 5A-B). In contrast, CD74 levels remained unchanged in LAN-1 whereas SH-SY5Y showed a significant decrease under all the experimental conditions (Additional Fig. 4A-B).

High levels of CXCR4 on the cell surface have been associated with intracellular activation of PI3K/AKT and MAPK/ERK signaling pathways [35]. We explored this possibility in our experimental setting. LAN-1 cells showed higher AKT phosphorylation (p-AKT) levels after 48 h exposure to CM-BM and CM-BM/NB under normoxia and hypoxia, whereas ERK phosphorylation (p-ERK) levels increased only in hypoxia (Fig. 5C). SH-SY5Y showed higher p-AKT and p-ERK in all CM, irrespective of oxygen levels (Fig. 5D).

These findings indicated that the BM-like experimental conditions increased the expression of CXCR4 on NB cell surface, and this resulted in the activation of oncogenic pathways such as PI3K/AKT and MAPK/ERK signaling.

### Neuroblastoma cells are selectively sensitive to the MIF inhibitor 4-IPP

**Fig. 6:**
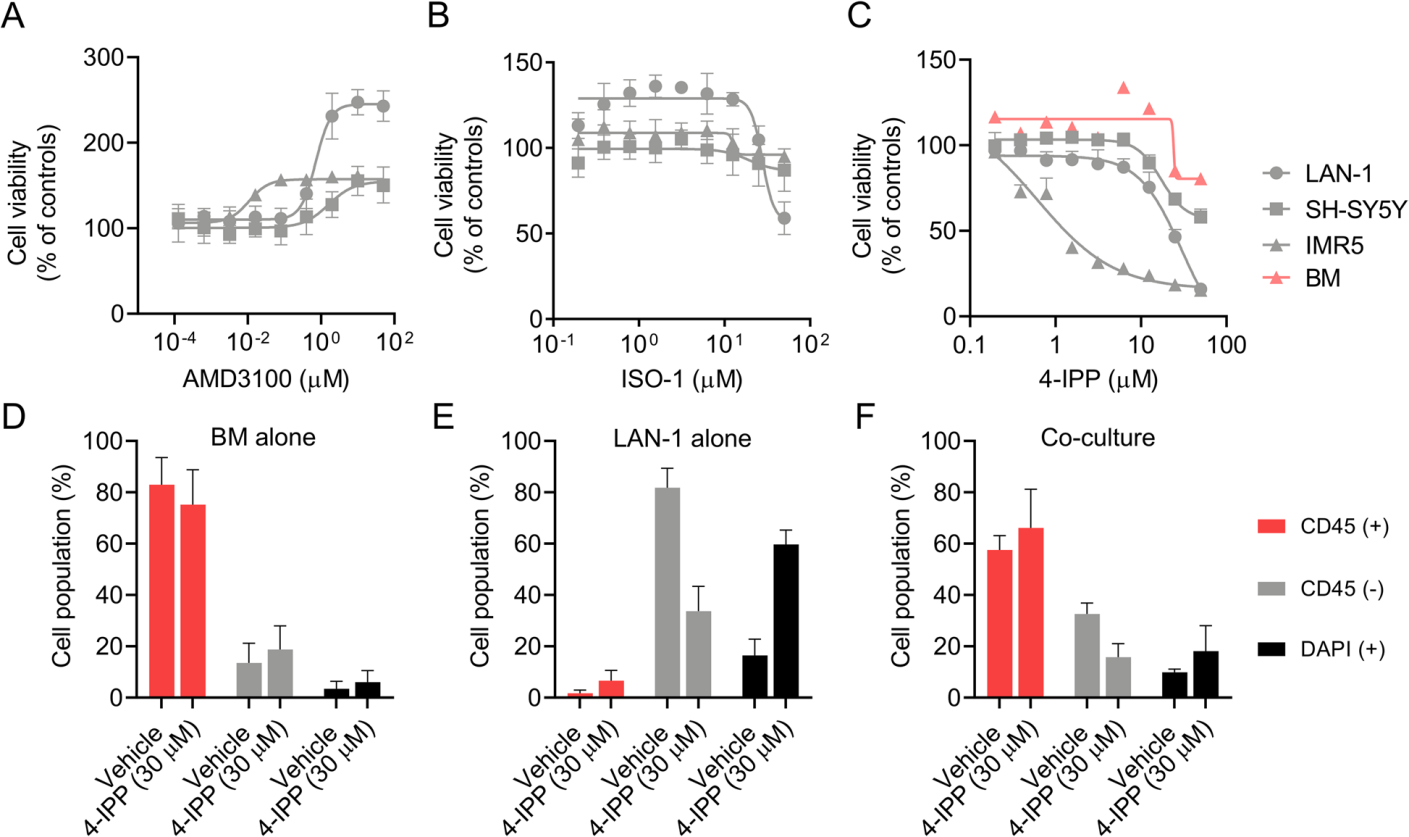
The activity of MIF inhibitors on neuroblastoma cell lines and BM primary cultures. Cytotoxic activity of **A)** AMD3100 (50-0.0001 μM), **B)** ISO-1 (50-0.2 μM) and **C)** 4-IPP (50-0.2 μM) in a panel NB cell lines and BM primary culture under regular conditions (*n =* 6). Flow cytometry quantification in bar graphs of 4-IPP activity on **D)** BM, **E)** LAN-1, and **F)** co-cultures LAN1:BM ratio (1:10) treated with vehicle or 4-IPP 30 μM after 72 h at regular conditions (*n =* 2)

To evaluated MIF and CXCR4 as potential mediators for NB progression within the BM niche, we explored different pharmacological approaches to target both proteins. First, we evaluated the efficacy of the CXCR4 antagonist AMD3100 (concentration range 50-0.0001 μM) against NB cell viability in regular in vitro cell culture conditions. We observed no significant effect on cell viability at low concentrations (below 1 μM) after 72 h, on the contrary, AMD3100 enhanced NB cellular viability at concentrations higher than 1 μM (Fig. 6A). Although in contrast with what we expected, similar findings have been reported in previous studies on pediatric solid tumors [36]. Thus, we focused on selective targeting of MIF using the antagonists ISO-1 and 4-IPP (4-iodo-6-phenylpyrimidine), which inhibits MIF dopachrome tautomerase activity by binding to its catalytic active site [30]. ISO-1 treatment (concentration range 50-0.2 μM) showed limited cytotoxic activity on cell viability (IC50 values higher than 50 μM; Fig. 6B), whereas 4-IPP (50-0.2 μM) showed concentration-dependent cytotoxicity against NB cells (LAN-1, SH-SY5Y, and IMR5) with a mean IC50 of 15.85 μM (±15.01; SD) after 72 h. Importantly, 4-IPP showed no cytotoxic effect on BM primary cell cultures (IC50 values higher than 50 μM; Fig. 6C). To determine whether the cytotoxic effects were selective against NB cells, we treated LAN-1 cells co-cultured with fresh patient-derived primary BM-MNCs cells with 30 μM of 4-IPP for 72 h in regular media. Cell population analysis by flow cytometry showed that 4-IPP diminished LAN-1 viability without affecting BM cells. The percentage of viable LAN-1 cells (CD45^−^/DAPI^−^) decreased to 16.8% after treatment, whereas no differences were observed in BM cells (CD45^+^/DAPI^−^); being percentages of 57.55 and 66.09% in control and treated cells, respectively (Fig. 6D-F, Additional Fig. 5). These findings showed that MIF inhibition with 4-IPP selectively affected NB cell viability without impairing patient-derived BM primary cells in co-culture in vitro models.

### MIF inhibitor impairs neuroblastoma aggressiveness in the bone marrow niche and reduces in vivo tumor growth

**Fig. 7:**
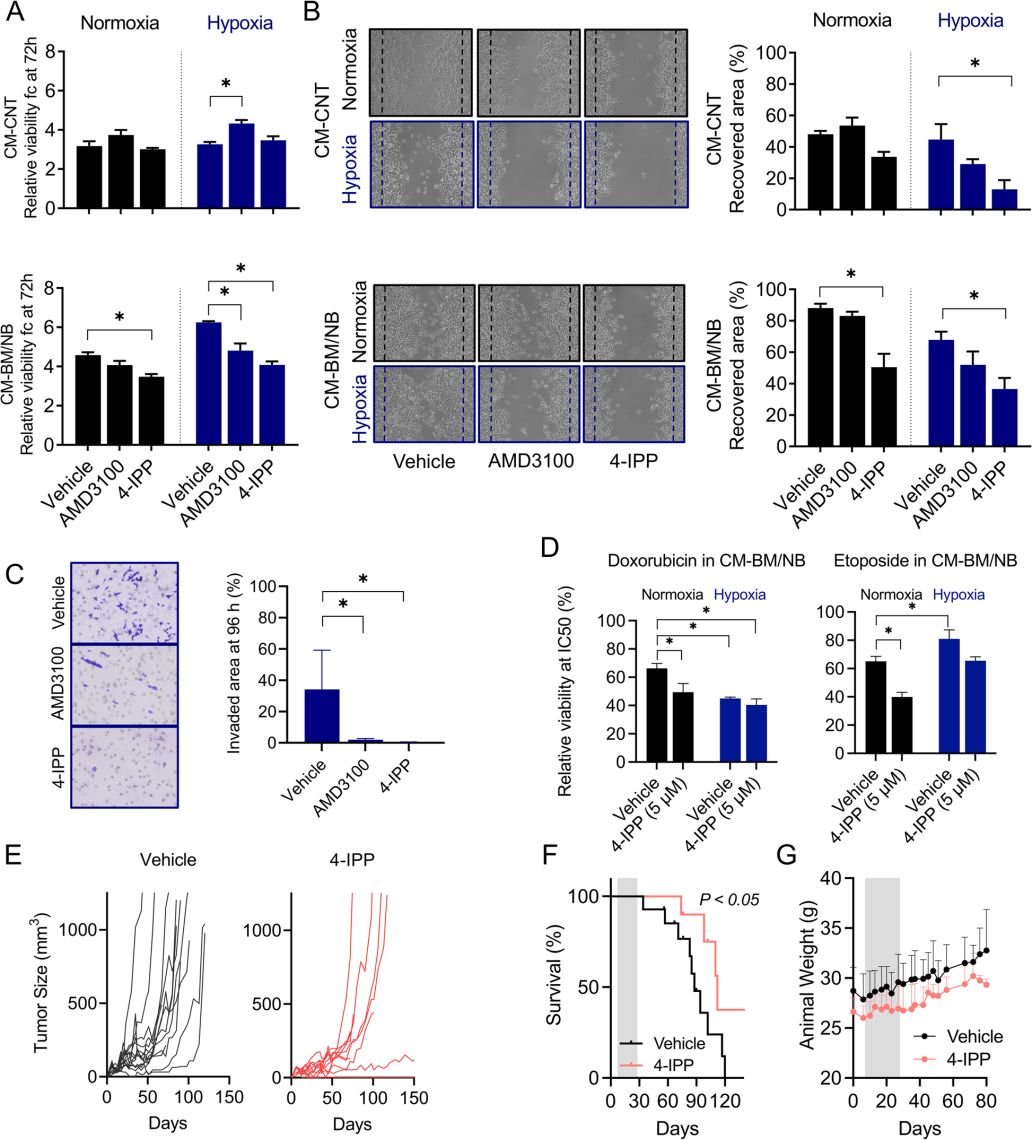
Impairing NB aggressive phenotypes with MIF and CXCR4 inhibitors. **A)** Cell viability after exposure to CM-CNT or CM-BM/NB and treatments (vehicle, 10 nM AMD3100 and 5 μM 4-IPP) at 72 h. Data is represented as a fold-change viability to time 0 h (*n =* 3). Significances were obtained after comparing vehicle vs treatments. One-way Anova Kruskal-Wallis test. **B)** Images (10x) and graph bar quantification of LAN-1 wound healing recovery under CM-CNT and CM-BM/NB normoxia and hypoxia (vehicle, 10 nM AMD3100 and 5 μM 4-IPP) at 24 h. Two-way Anova Sidaks’s multiple comparison test. **C)** Images and graph bars of invaded cells with 5 μM 4-IPP or 10 nM AMD3100, under CM-BM/NB normoxia (black) and hypoxia (blue) at 96 h. One-way Anova Kruskal-Wallis test. * *P <* 0.05. **D)** Cytotoxicity of chemotherapy compounds, doxorubicin and etoposide, in LAN-1 cells cultured with CM-BM/NB at 72 h together with 5 μM of 4-IPP or vehicle. Bar graph with relative viability at CM-BM/NB for doxorubicin and etoposide IC50. Normoxia (black), hypoxia (blue). Two-way Anova, Dunnett’s multiple comparison test. (*n =* 3) (*) *P <* 0.05. **E)** Individual tumor growth in vehicle and 4-IPP treated group. **F)** Tumor survival curves for each group. Events were reported when tumor size reached 1000 mm^3^. **G)** Body weight variation of mice during treatment (grey area) and follow-up

Based on our findings and the current knowledge about MIF signaling in can﻿cer [18, 30, 37], we investigated whether the pro-tumorigenic effect of our CM was related to MIF signaling activity, potentially through the CXCR4 receptor. To this end, we explored whether treatment with 4-IPP or AMD3100 could revert the oncogenic features promoted by CM-BM/NB and hypoxia. At concentrations that did not affect cell viability (below IC50 values: 5 μM for 4-IPP, and 10 nM for AMD3100) in regular medium conditions, both drugs decreased LAN-1 cell viability after 72 h of treatment in the presence of CM-BM/NB both under normoxia and hypoxia conditions, being statistically significant for 4-IPP (*P* < 0.05) (Fig. 7A). No differences in cell viability were observed when 4-IPP was added to LAN-1 cells cultured in CM-CNT, either under normoxia or hypoxia (Fig. 7A, and CM-NB, CM-BM in Additional Fig. 6A). Likewise, LAN-1 exposed to 5 μM of 4-IPP showed less migratory capacity in wound healing assays as compared to vehicle-treated controls in both hypoxia and normoxia (*P* < 0.05), whereas no significant differences were observed with AMD3100 (10 nM) (Fig. 7B). In transwell chamber assays, both drugs showed a clear inhibition of LAN-1 cell chemoattraction towards CM-BM/NB under hypoxic conditions after 24 h of treatment (percentage of the invaded area of 34.1 ± 25, 1.9 ± 0.78, and 0.43 ± 0.19 vehicle, AMD3100, and 4-IPP, respectively; mean and SD (*P *< 0.05); Fig. 7C).

MIF inhibitors have been reported to potentially improve chemotherapy cytotoxicity in vitro. Thus, we explored whether MIF inhibition with 4-IPP could modulate drug resistance observed in LAN-1 cultured in CM-BM/NB and restore drug sensitivity to doxorubicin and etoposide. To this end, we combined 4-IPP (5 μM) with doxorubicin and etoposide at concentrations close to IC50 values (30 nM and 415 nM, respectively). The addition of 4-IPP increased the concentration-dependent cytotoxicity of both doxorubicin and etoposide, significantly reducing viability at IC50 values in normoxia and hypoxia (*P < 0.01*) (Fig. 7D; CM-CNT Additional Fig. 6B). These findings suggested MIF inhibition could impair NB expansion in the BM niche.

We thus explored if MIF inhibition could impair NB cell tumor growth in vivo using LAN-1 xenograft models in athymic nude mice. We envisaged MIF inhibition as a potential approach for the treatment of recurrent high-risk NB which usually occurs within the BM niche from minimal residual disease (MRD). We tested the antitumor effect of 4-IPP (80 mg/kg) in an MRD scenario where animal treatments were initiated 7 days after subcutaneous injection of 1.5 × 10^6^ LAN-1 cells per flank. After 4 weeks of treatment, animals were followed up and tumor growth was measured every 3-4 days until endpoint criteria. Treatment with 4-IPP significantly delayed tumor growth and prevented tumor formation in 1 out of 10 inoculated tumors as compared to controls (100% of engraftment) (Fig. 7E). 4-IPP improved animal survival with a benefit of 24 days (median survival of 88 days vehicle group vs. 112 days 4-IPP; *P* < 0.05) (Fig. 7F). No adverse side effects on the animal such as physical symptoms, decreased activity, or body weight loss were observed in the 4-IPP treatment group (Fig. 7G). Taken together, our results showed that inhibition of MIF affected tumors by delaying initial growth, however, treatment with 4-IPP was not enough to inhibit the aggressive behavior of NB cells.

## Discussion

In the present study, we have identified macrophage migration inhibitory factor (MIF) as a key inflammatory cytokine potentially involved in Bone Marrow (BM) infiltration in patients with high-risk neuroblastoma (NB). At the moment of diagnosis, 50% of NB patients show disease dissemination to distal sites [1]. Among these, BM represents the most common site for dissemination, and its infiltration at diagnosis and relapse is a hallmark of NB stage 4 disease [4]. Therefore, the unique biological properties of the BM environment make it a preferential niche for NB dissemination. Recently, a multi-omics study showed that BM remodeling in presence of NB infiltration, prone the environment towards a pro-inflammatory and immunosuppressive state [37]. However, the biological mechanisms underlying NB growth and invasion within the BM niche are still largely unknown. In this study, we have used in vitro cell culture conditions that recapitulated some specific features found in the BM microenvironment of NB patients, such as the cell-derived secreted factors and low oxygen levels. Diverse strategies to mimic BM in vitro have been reported previously, including multiple cellular co-cultures or extracellular matrix-based 3D cultures [38*,* 39]. Although our model only partly represents the complex environment of the BM-niche, it enabled us to identify MIF as the principal cytokine secreted by NB cells, that persisted when cultured with patient-derived BM aspirates, mimicking an infiltrated BM environment.

MIF is a pleiotropic cytokine that plays a key role in several diseases, including cancer [40]. In NB, MIF expression has been reported to be associated with tumor progression, immune evasion, inflammation, expression of pro-angiogenic factors, and *MYCN* oncogene [20*,* 21*,* 41]. By analyzing RNA sequencing data, we identified significant upregulation of *MIF* and *CXCR4* expression in high-risk disseminated NB tumors, which was significantly associated with patient poor outcomes. Interestingly, we also identified high expression of *MIF* in BM-derived disseminated NB tumor cells, and thus associated with NB infiltration. These findings suggested NB cellularity as the main source of MIF within the BM disseminated niche, highlighting a potential role of MIF in NB survival and tumor progression in the BM.

Although MIF is known to be secreted by NB cell lines, its role in the maintenance and expansion of NB cells in the BM has never been studied. Furthermore, the MIF-CXCR4 ligand-receptor axis has never been explored in NB, whereas the role of the well-studied axis CXCL12/CXCR4 is still controversial [18*,* 42*,* 43]. The increment of the chemokine receptor CXCR4 on the NB cell surface has been associated with aggressive tumor behavior such as cell proliferation, migration, and invasion. These features have generally been related to the CXCL12/CXCR4 signaling pathway [44*,* 45]. In our BM-CM, the chemokine ligand CXCL12 was undetectable. Nonetheless, we detected increased levels of CXCR4 on the surface of NB cells when exposed to BM-derived CMs (CM-BM/NB) and hypoxia. This was associated with higher NB cell viability, greater migration-invasion capacity, and activation of oncogenic pathways such as PI3K/AKT and MAPK/ERK signaling pathways, potentially contributing to the survival and growth of NB cell lines. These aggressive phenotypes were not dependent on the presence of the CXCL12 cytokine in the extracellular environment but could be, in part, mediated by MIF/CXCR4 signaling [19]. The MIF/CXCR4 axis has been described in different cancer types as a critical autocrine pathway linked to tumor progression [46–49]. MIF/CXCR4 signaling promotes neovascularization, migration, and invasion phenotypes in colorectal cancer, non-small cell lung cancer, and glioblastoma [46–48]. Besides, extracellular MIF has been identified in a chick developmental model as a potent chemoattractant for trunk neural crest cells, plausible NB progenitor cells [50, 51]. MIF also interacts with CD74, a known chaperone of the invariant chain of major histocompatibility complex class II (MHC II) [52]. In our BM-based experimental conditions, NB cell surface levels of CD74 did not vary, suggesting a potential minor role of this pleiotropic receptor in the response of NB cells to the BM environment. However, CD74 has alternative MIF-dependent and non-dependent roles, thus its potential role in NB biology cannot be dismissed and needs to be further explored.

Evidence of the critical pro-tumorigenic role of the BM microenvironment was observed on NB cells cultured in the presence of cell-secreted factors, in the form of CM, and hypoxia. When exposed to our BM-derived CMs, increased cell viability was reported in the NB cell lines derived from stage 4 patients, LAN-1, SH-SY5Y, and the patient derived cell line HSJD-NB01. However, this response was not observed when using a cell line derived from an abdominal mass (IMR5) with unknown dissemination status [53]. On the other hand, hypoxia promoted the viability of the *MYCN*-amplified cells LAN-1, IMR5, and HSJD-NB01, while not affecting SH-SY5Y, lacking *MYCN* amplification. A combinatorial effect between MYCN and HIF-1α, has been reported to contribute to NB aggressiveness, particularly in *MYCN* amplified cells [54]. Our experimental conditions significantly increased migration capacity, while promoting invasion and chemoresistance. Furthermore, when we injected subcutaneously BM cells mixed with NB cells in an athymic nude mice model, tumor engraftment was significantly enhanced. These findings underscored the relevance of cell-derived secreted factors found in the BM microenvironment for tumor aggressive phenotypes. Different studies had previously described that NB cell lines derived from metastatic lesions displayed increased tumorigenicity in vitro and in vivo in the presence of purified human BM MSCs [55, 56]. The BM environment thus contributes to NB tumorigenicity, generating a favorable microenvironment that benefits rapid NB tumor development.

MIF signaling has been previously related to drug resistance in different tumor types, including NB and other developmental tumors [20*, *49*, *57*, *58]. We were able to impair NB malignant behavior induced by our BM-conditions and improve drug response in vitro and in vivo by adding sub-lethal concentrations of the MIF inhibitor 4-IPP. In agreement with our results, Zheng et al. showed that 4-IPP enhanced melphalan cytotoxicity in multiple myeloma, a BM infiltrating tumor with high MIF dependency [49]. Our study showed that pharmacological targeting of MIF with 4-IPP delayed NB tumor growth, improving overall survival in LAN-1 xenograft models. These results are consistent with previous studies reporting a reduction of tumor growth in a xenograft model of NB cell lines transfected with an antisense MIF expression vector and delayed tumor growth in a melanoma syngeneic model treated with 4-IPP [41, 58].

## Conclusions

In conclusion, our findings provide new insights on the contribution of the BM microenvironment to NB disseminated disease. Based on our BM-like conditions the relationship between the BM microenvironment and NB cells appears to be mediated, in part, by the MIF-CXCR4 signaling axis. Pharmacological inhibition of MIF needs to be explored in more comprehensive preclinical studies since it could represent a therapeutic option for patients with high-risk NB.

## Supplementary Information

**Additional file 1: Additional Fig. 1**: Gene expression in neuroblastoma tumors and cell lines. The figure includes correlation study and survival analysis from NB patient datasets and flow cytometry analysis from neuroblastoma cell lines. **Additional Fig. 2**: Validation of in vitro hypoxia cytometry. **Additional Fig. 3**: Effect of human recombinant MIF and siCXCR4 in neuroblastoma cell lines. **Additional Fig. 4**: Membrane CD74 levels by flow cytometry. **Additional Fig. 5**: Flow cytometry density plots of 4-IPP activity. **Additional Fig. 6**: LAN-1 viability exposed to CM-NB, CM-BM and treated with AMD-3100 and 4-IPP. LAN-1 response to chemotherapeutic agents when exposed to CM-CNT and treated with 4-IPP. **Additional Table 1**: Bone marrow samples. **Additional Table 2**: Primer list, and **Additional Table 3**: Antibody list.

## Data Availability

Publicly available data used in this manuscript can be obtained in the Gene Expression Omnibus repository web (www.ncbi.nlm.nih.gov/geo) under the reference: GSE94035 and GSE62564.

## Abbreviations

NB: Neuroblastoma
BM: Bone Marrow
INSS: International Neuroblastoma Staging System
MSC: Mesenchymal Stem Cell
HSC: Hematopoietic Stem Cell
CM: Conditioned Media
Nx: Normoxia
Hx: Hypoxia
MRD: Minimal Residual Disease
OS: Overall Survival
MNC: Mononuclear cell
SD: Standard Deviation
SC: Sub-cutaneous
FFPE: Formalin-fixed paraffin-embedded
