# Supplementary material for: MIF/CXCR4 signaling axis contributes to survival, invasion, and drug resistance of metastatic neuroblastoma cells in the bone marrow microenvironment
Source: BMC Cancer. 2022 Jun 17;22:669. doi: 10.1186/s12885-022-09725-8 (PMC9206243; doi:10.1186/s12885-022-09725-8)
Supplement: Supplementary file 1 — Additional file 1: Additional Fig. 1: Gene expression in neuroblastoma tumors and cell lines. The figure includes correlation study and survival analysis from NB patient datasets and flow cytometry analysis from neuroblastoma cell lines. Additional Fig. 2: Validation of in vitro hypoxia cytometry. Additional Fig. 3: Effect of human recombinant MIF and siCXCR4 in neuroblastoma cell lines. Additional Fig. 4: Membrane CD74 levels by flow cytometry. Additional Fig. 5: Flow cytometry density plots of 4-IPP activity. Additional Fig. 6: LAN-1 viability exposed to CM-NB, CM-BM and treated with AMD-3100 and 4-IPP. LAN-1 response to chemotherapeutic agents when exposed to CM-CNT and treated with 4-IPP. Additional Table 1: Bone marrow samples. Additional Table 2: Primer list, and Additional Table 3: Antibody list. [file 12885_2022_9725_MOESM1_ESM.zip › Additional Table 1.pdf]

Additional Table 1

ID	MRD (*PHOX2B*)	INSS	Treatment
BMNB_32	no detected	na	Prior to Consolidation
BMNB_35	no detected	4	Prior to Rescue
BMNB_38	no detected	na	Post-Consolidation
BMNB_39	no detected	na	Prior to Consolidation
BMNB_41	no detected	4	Consolidation
BMNB_42	<1:30000	na	Consolidation
BMNB_43	no detected	4	Post-Consolidation
BMNB_45	no detected	3	Consolidation
BMNB_46	no detected	4	Consolidation
BMNB_47	<1:18000	na	Consolidation
BMNB_51	no detected	4	Post-Consolidation
BMNB_52	no detected	na	Post-Consolidation
BMNB_55	no detected	na	Consolidation
BMNB_56	<1:100000	na	Prior to Rescue
BMNB_57	no detected	4	Prior to Consolidation
BMNB_65	no detected	na	Consolidation
BMNB_68	no detected	na	Post-Consolidation
BMNB_75	no detected	na	Prior Consolidation
BMNB_81	no detected	4	Consolidation
BMNB_83	no detected	4	Rescue

Patient-derived BM aspirates from NB patients.

MRD: minimal residual disease

INSS: International Neuroblastoma Staging System

na: non-available
