# Supplementary material for: MIF/CXCR4 signaling axis contributes to survival, invasion, and drug resistance of metastatic neuroblastoma cells in the bone marrow microenvironment
Source: BMC Cancer. 2022 Jun 17;22:669. doi: 10.1186/s12885-022-09725-8 (PMC9206243; doi:10.1186/s12885-022-09725-8)
Supplement: Supplementary file 1 — Additional file 1: Additional Fig. 1: Gene expression in neuroblastoma tumors and cell lines. The figure includes correlation study and survival analysis from NB patient datasets and flow cytometry analysis from neuroblastoma cell lines. Additional Fig. 2: Validation of in vitro hypoxia cytometry. Additional Fig. 3: Effect of human recombinant MIF and siCXCR4 in neuroblastoma cell lines. Additional Fig. 4: Membrane CD74 levels by flow cytometry. Additional Fig. 5: Flow cytometry density plots of 4-IPP activity. Additional Fig. 6: LAN-1 viability exposed to CM-NB, CM-BM and treated with AMD-3100 and 4-IPP. LAN-1 response to chemotherapeutic agents when exposed to CM-CNT and treated with 4-IPP. Additional Table 1: Bone marrow samples. Additional Table 2: Primer list, and Additional Table 3: Antibody list. [file 12885_2022_9725_MOESM1_ESM.zip › Additional Table 2.pdf]

Supplementary Table 2

Gene	Fw (5'→3')	Rv (5'→3')
HIF-1 α	TTCCAGTTACGTTCCTTCGATCA	TTTGAGGACTTGCGCTTTCA
HIF-2 α	GTGCTCCCACGGCCTGTA	TTGTCACACCTATGGCATATCACA
VEGF	AGGAGGAGGGCAGAATCATCA	CTCGATTGGATGGCAGTAGCT
GLUT1	GCCATACTCATGACCATCGC	AGCTCCTCGGGTGTCTTATC
OCT4	GAAACCCACACTGCAGATCA	CGGTTACAGAACCACACTCG
YWHAZ	ACTTTTGGTACATTGTGTGGCTTCAA	CCGCCAGGACAAACCAGTAT
