# Supplementary material for: MIF/CXCR4 signaling axis contributes to survival, invasion, and drug resistance of metastatic neuroblastoma cells in the bone marrow microenvironment
Source: BMC Cancer. 2022 Jun 17;22:669. doi: 10.1186/s12885-022-09725-8 (PMC9206243; doi:10.1186/s12885-022-09725-8)
Supplement: Supplementary file 1 — Additional file 1: Additional Fig. 1: Gene expression in neuroblastoma tumors and cell lines. The figure includes correlation study and survival analysis from NB patient datasets and flow cytometry analysis from neuroblastoma cell lines. Additional Fig. 2: Validation of in vitro hypoxia cytometry. Additional Fig. 3: Effect of human recombinant MIF and siCXCR4 in neuroblastoma cell lines. Additional Fig. 4: Membrane CD74 levels by flow cytometry. Additional Fig. 5: Flow cytometry density plots of 4-IPP activity. Additional Fig. 6: LAN-1 viability exposed to CM-NB, CM-BM and treated with AMD-3100 and 4-IPP. LAN-1 response to chemotherapeutic agents when exposed to CM-CNT and treated with 4-IPP. Additional Table 1: Bone marrow samples. Additional Table 2: Primer list, and Additional Table 3: Antibody list. [file 12885_2022_9725_MOESM1_ESM.zip › Additional Table 3.pdf]

Supplementary Table 3

Antibody	Use	Working dilution	Ref	Company
Phospho-p44/42 (Erk1/2) (Thr202/Tyr204)	WB	1:1000	4370	Cell Signaling Technology
p44/42 MAPK (Erk1/2)	WB	1:1000	4695	Cell Signaling Technology
Phospho-Akt (Ser473)	WB	1:1000	4060	Cell Signaling Technology
AKT	WB	1:1000	9272	Cell Signaling Technology
HIF-1α	WB	1:500	51608	Abcam
CXCR4	WB	1:1000	CSB-PA006254YA01HU	Acusabio
MIF	WB	1:1000	SM6013S	Origene
gamma-Tubulin	WB	1:30000	T6557	Sigma
IRDye® 680RD α-Rabbit	WB	1:5000	926-68071	Li-COR
IRDye® 800CW α-Rabbit	WB	1:5000	926-32211	Li-COR
IRDye® 680RD α-Mouse	WB	1:5000	926-68070	Li-COR
IRDye® 800RD α-Mouse	WB	1:5000	926-32210	Li-COR
CXCR4-PE	FC	1:10	FAB170P	R&D Systems
CD74-Alexa467	FC	0.2 µg/mL	FAB35901R	R&D Systems
CD45-V500	FC	2.5 µg/mL	655873	Becton Dickinson
CD11b-PE	FC	5 µg/mL	333142	Becton Dickinson
CD34 PerCP	FC	1.25 µg/mL	345803	Becton Dickinson
HLA-DR PerCP	FC	2.5 µg/mL	347402	Becton Dickinson
CD19-PeCy7	FC	2.5 µg/mL	341113	Becton Dickinson
CD123-APC	FC	2.5 µg/mL	658171	Becton Dickinson
CD117-APC	FC	2.5 µg/mL	333233	Becton Dickinson
CD90-PE	FC	1:20	130-097-932	Milteny Biotech
CD105-FITC	FC	1:20	130-098-778	Milteny Biotech
CD45-PE	FC	1:50	130-110-632	Milteny Biotech
hNu	IHC	1:200	MAB4383	Merck Millipore
Ki-67	IHC	pre-diluted	PA0230	Leica Systems
