# Supplementary material for: MIF/CXCR4 signaling axis contributes to survival, invasion, and drug resistance of metastatic neuroblastoma cells in the bone marrow microenvironment
Source: BMC Cancer. 2022 Jun 17;22:669. doi: 10.1186/s12885-022-09725-8 (PMC9206243; doi:10.1186/s12885-022-09725-8)

Sample		
Sample	1	SH-SY5Y 48h Nx CNT
	2	SH-SY5Y 48h Nx NB
	3	SH-SY5Y 48h Nx BM
	4	SH-SY5Y 48h Nx BM/NB
	5	SH-SY5Y 48h Hx CNT
	6	SH-SY5Y 48h Hx NB
	7	SH-SY5Y 48h Hx BM
	8	SH-SY5Y 48h Hx BM/NB

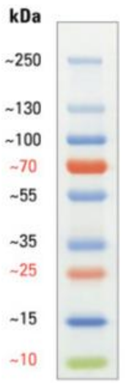

Figure 5 D - Cropped

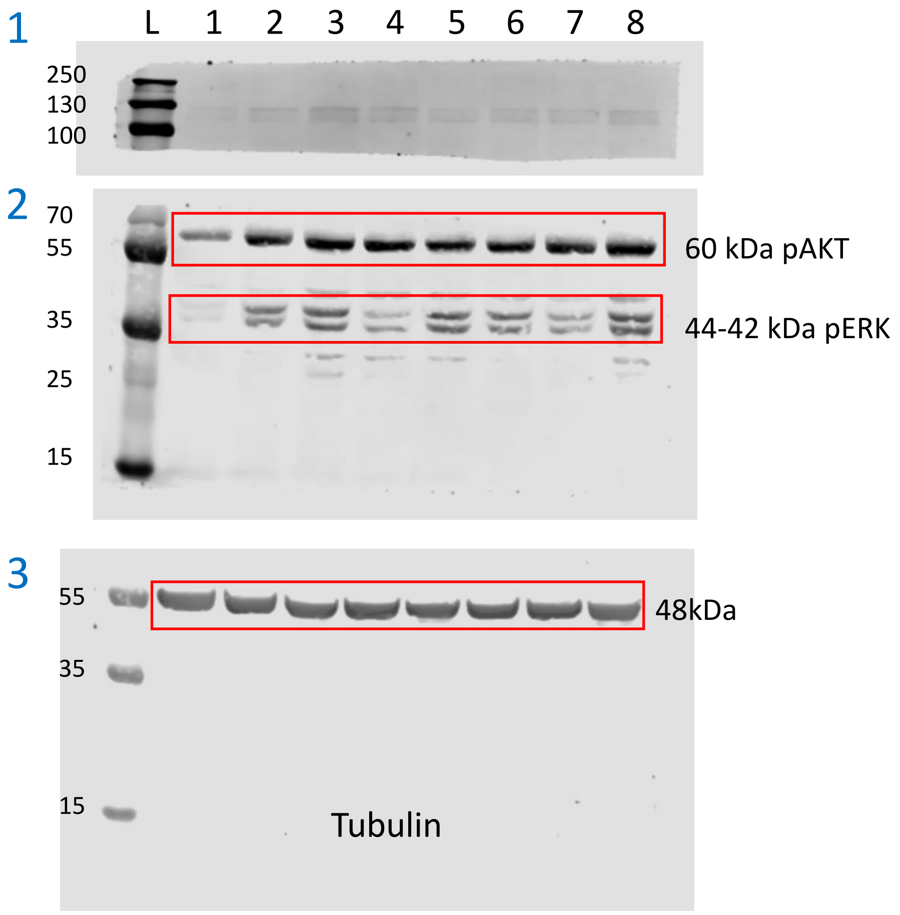

Figure 5 D – Uncropped

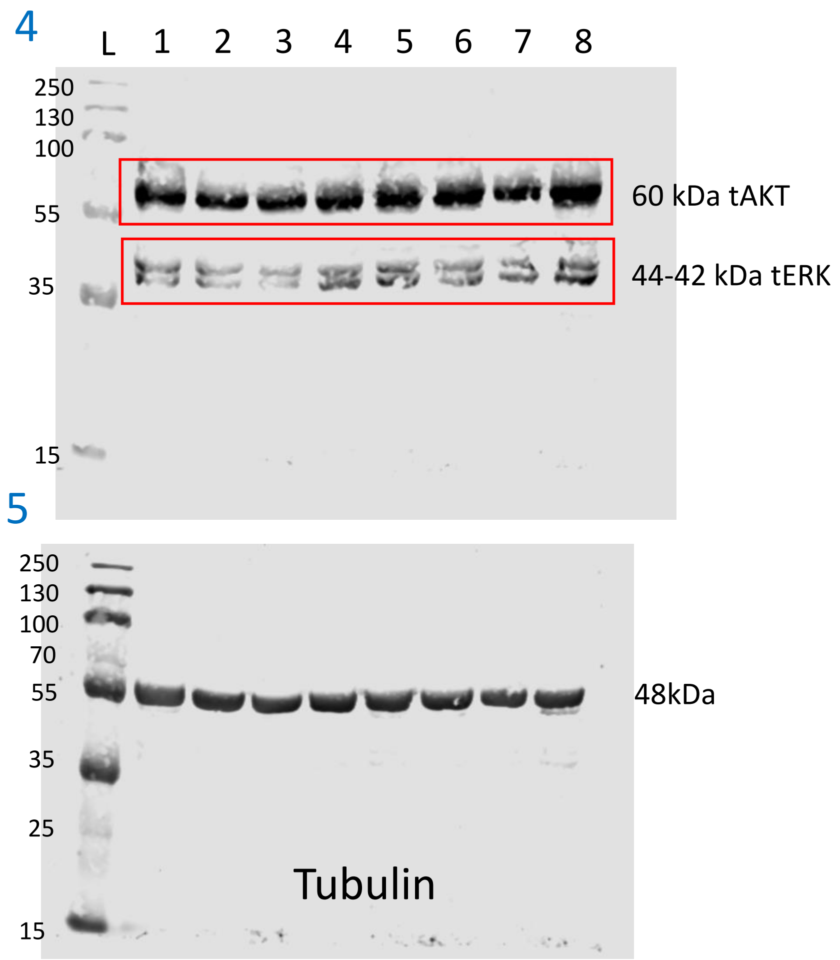

Gel	1	LAN-1 48h Nx CNT
	2	LAN-1 48h Nx NB
	3	LAN-1 48h Nx BM
	4	LAN-1 48h Nx BM/NB
	5	LAN-1 48h Hx CNT
	6	LAN-1 48h Hx NB
	7	LAN-1 48h Hx BM
	8	LAN-1 48h Hx BM/NB

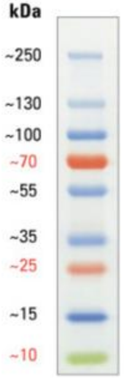

Figure 5 C - Cropped

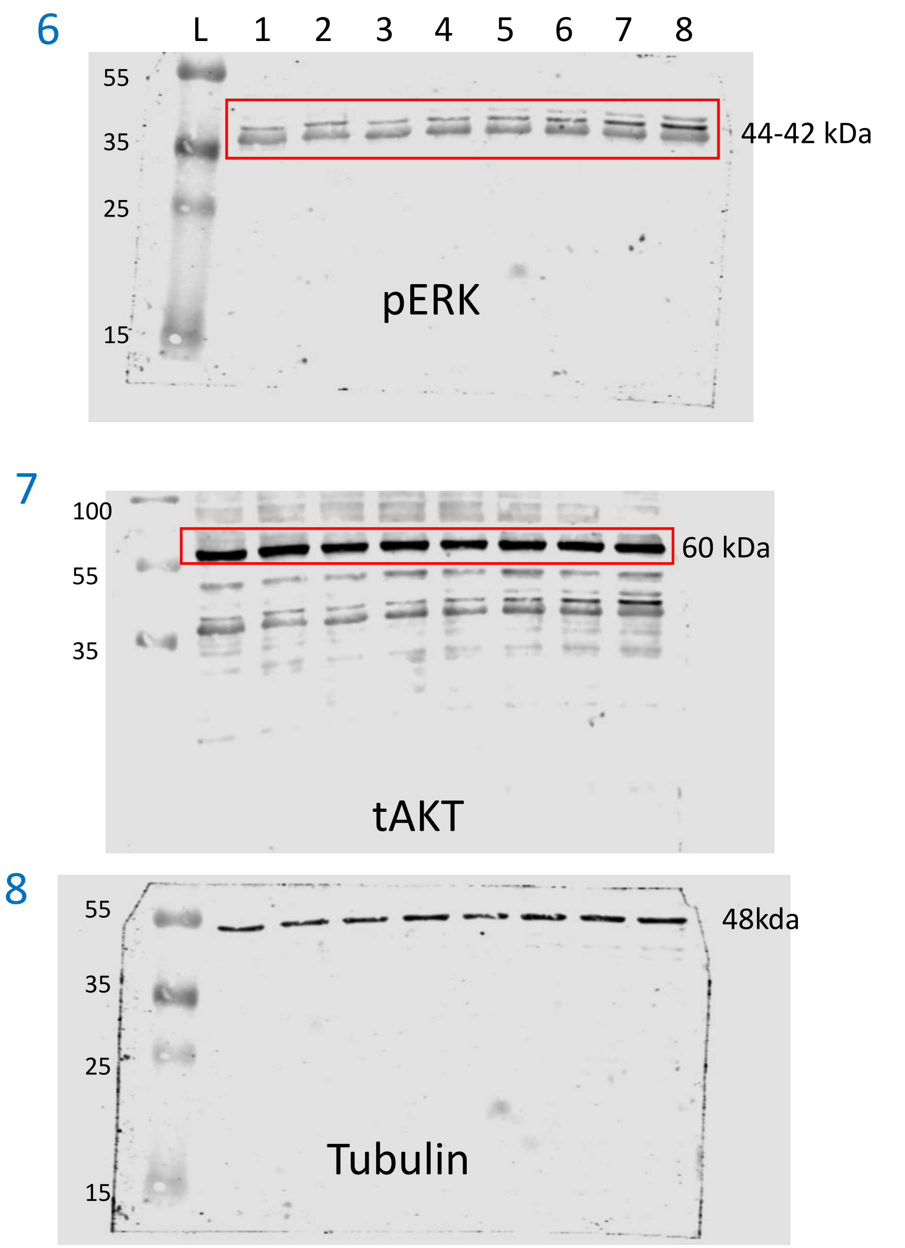

Figure 5 C – Uncropped

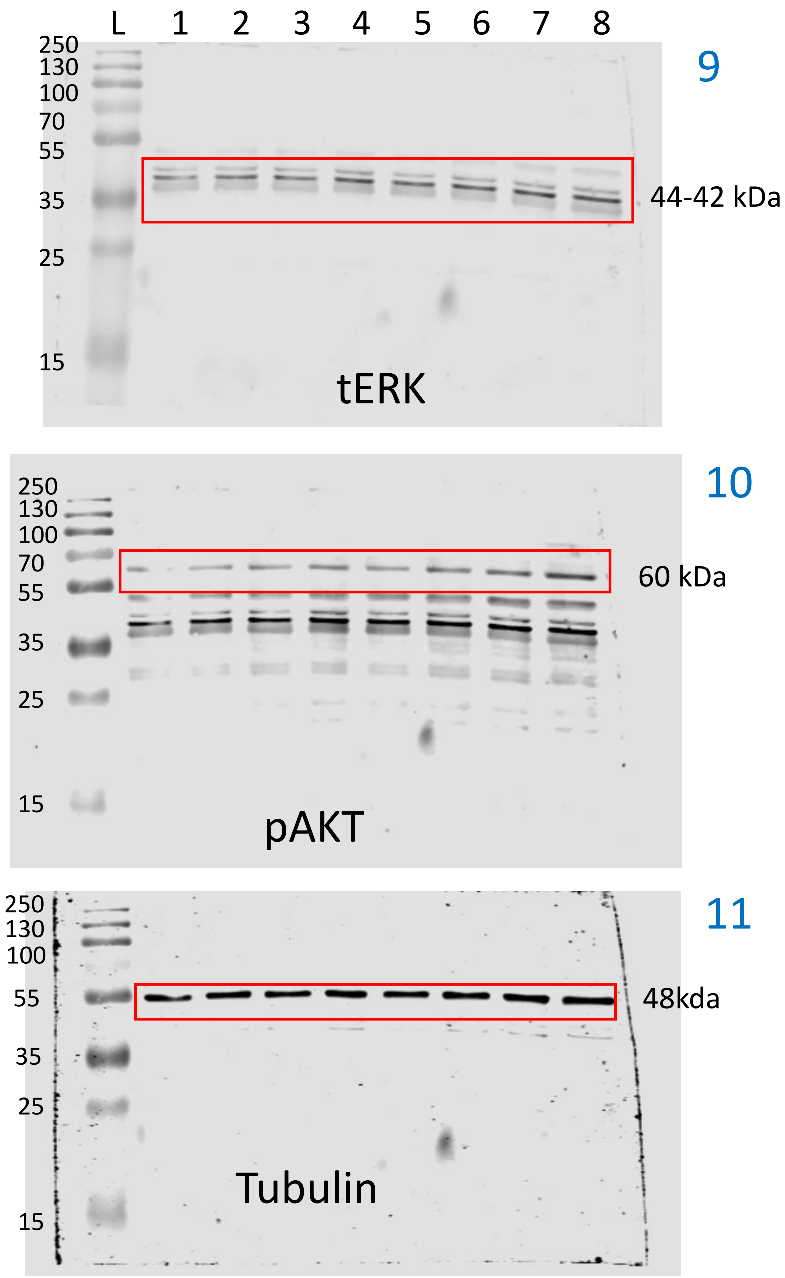

sample	1	LAN-1 Nx 0 $CoCl_2$
	2	LAN-1 Nx 30 µM $CoCl_2$
	3	LAN-1 Nx 150 µM $CoCl_2$
	4	LAN-1 Hx 1% O_2
	5	LAN-1 Hx 1% O_2

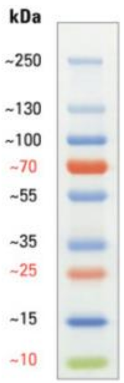

Figure S2 A – Uncropped

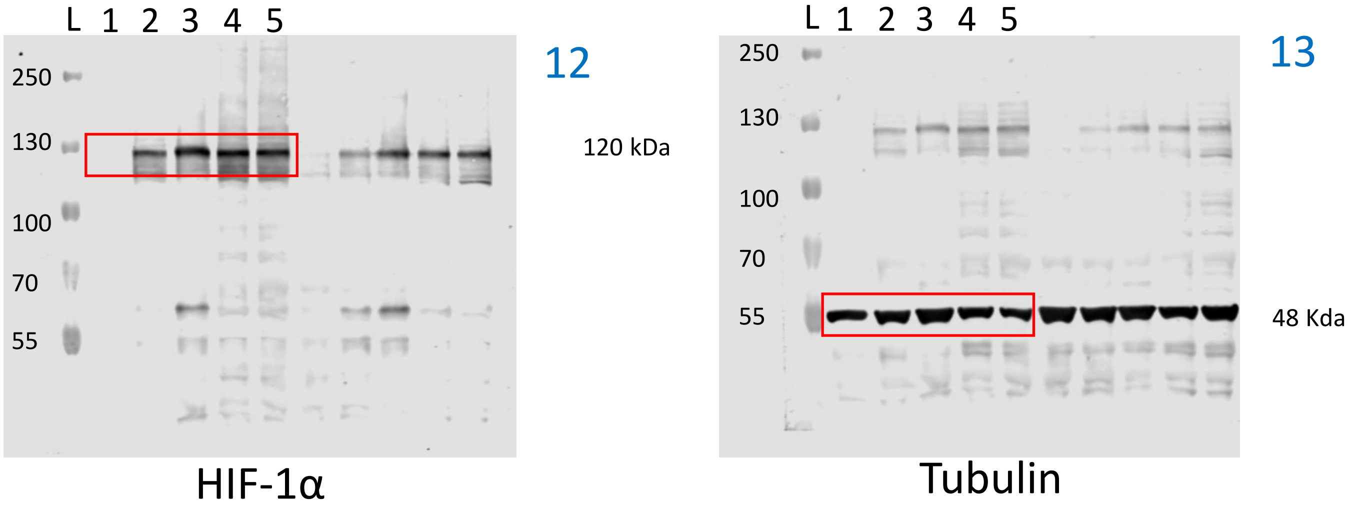

Figure S3 B – Uncropped

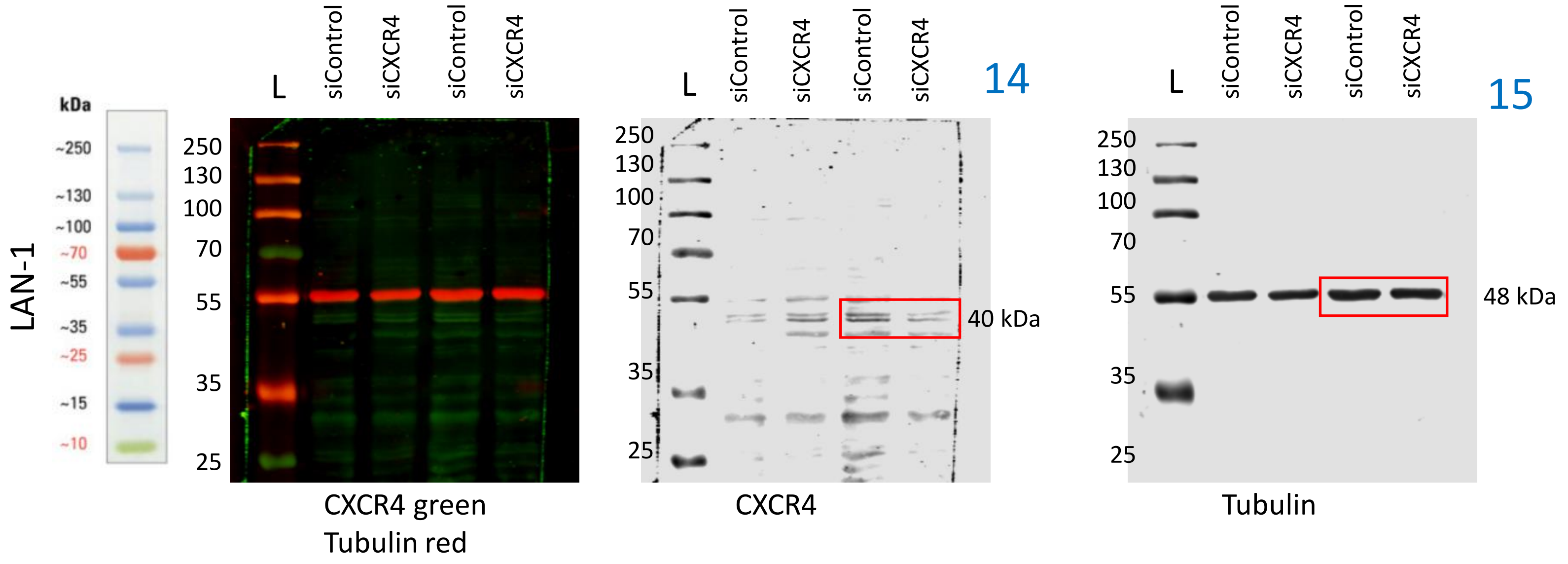

Figure S3 C – Uncropped

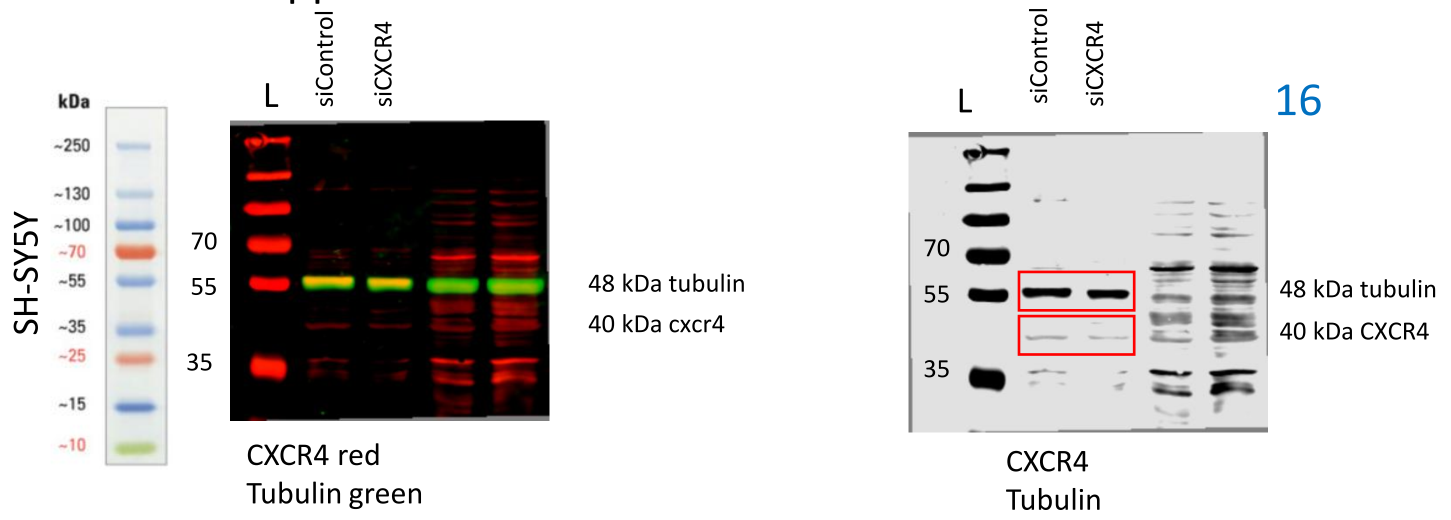

Figure 3S B and C – Uncropped

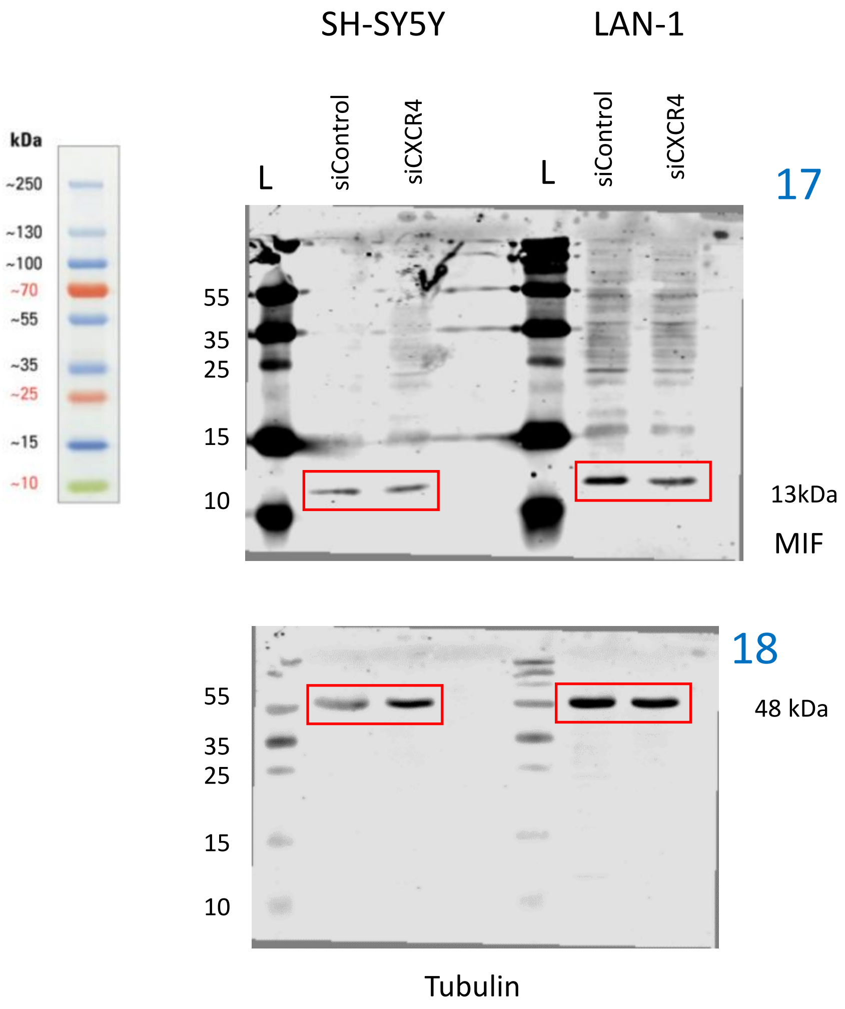

Supplement: Supplementary file 1 — Additional file 1: Additional Fig. 1: Gene expression in neuroblastoma tumors and cell lines. The figure includes correlation study and survival analysis from NB patient datasets and flow cytometry analysis from neuroblastoma cell lines. Additional Fig. 2: Validation of in vitro hypoxia cytometry. Additional Fig. 3: Effect of human recombinant MIF and siCXCR4 in neuroblastoma cell lines. Additional Fig. 4: Membrane CD74 levels by flow cytometry. Additional Fig. 5: Flow cytometry density plots of 4-IPP activity. Additional Fig. 6: LAN-1 viability exposed to CM-NB, CM-BM and treated with AMD-3100 and 4-IPP. LAN-1 response to chemotherapeutic agents when exposed to CM-CNT and treated with 4-IPP. Additional Table 1: Bone marrow samples. Additional Table 2: Primer list, and Additional Table 3: Antibody list. [file 12885_2022_9725_MOESM1_ESM.zip › WB supplementary.pdf]
