# Supplementary figures and images for: MIF/CXCR4 signaling axis contributes to survival, invasion, and drug resistance of metastatic neuroblastoma cells in the bone marrow microenvironment
Source: BMC Cancer. 2022 Jun 17;22:669. doi: 10.1186/s12885-022-09725-8 (PMC9206243; doi:10.1186/s12885-022-09725-8)

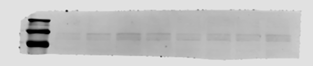

Supplement: Supplementary file 1 — Additional file 1: Additional Fig. 1: Gene expression in neuroblastoma tumors and cell lines. The figure includes correlation study and survival analysis from NB patient datasets and flow cytometry analysis from neuroblastoma cell lines. Additional Fig. 2: Validation of in vitro hypoxia cytometry. Additional Fig. 3: Effect of human recombinant MIF and siCXCR4 in neuroblastoma cell lines. Additional Fig. 4: Membrane CD74 levels by flow cytometry. Additional Fig. 5: Flow cytometry density plots of 4-IPP activity. Additional Fig. 6: LAN-1 viability exposed to CM-NB, CM-BM and treated with AMD-3100 and 4-IPP. LAN-1 response to chemotherapeutic agents when exposed to CM-CNT and treated with 4-IPP. Additional Table 1: Bone marrow samples. Additional Table 2: Primer list, and Additional Table 3: Antibody list. [file 12885_2022_9725_MOESM1_ESM.zip › blot 1.tif]

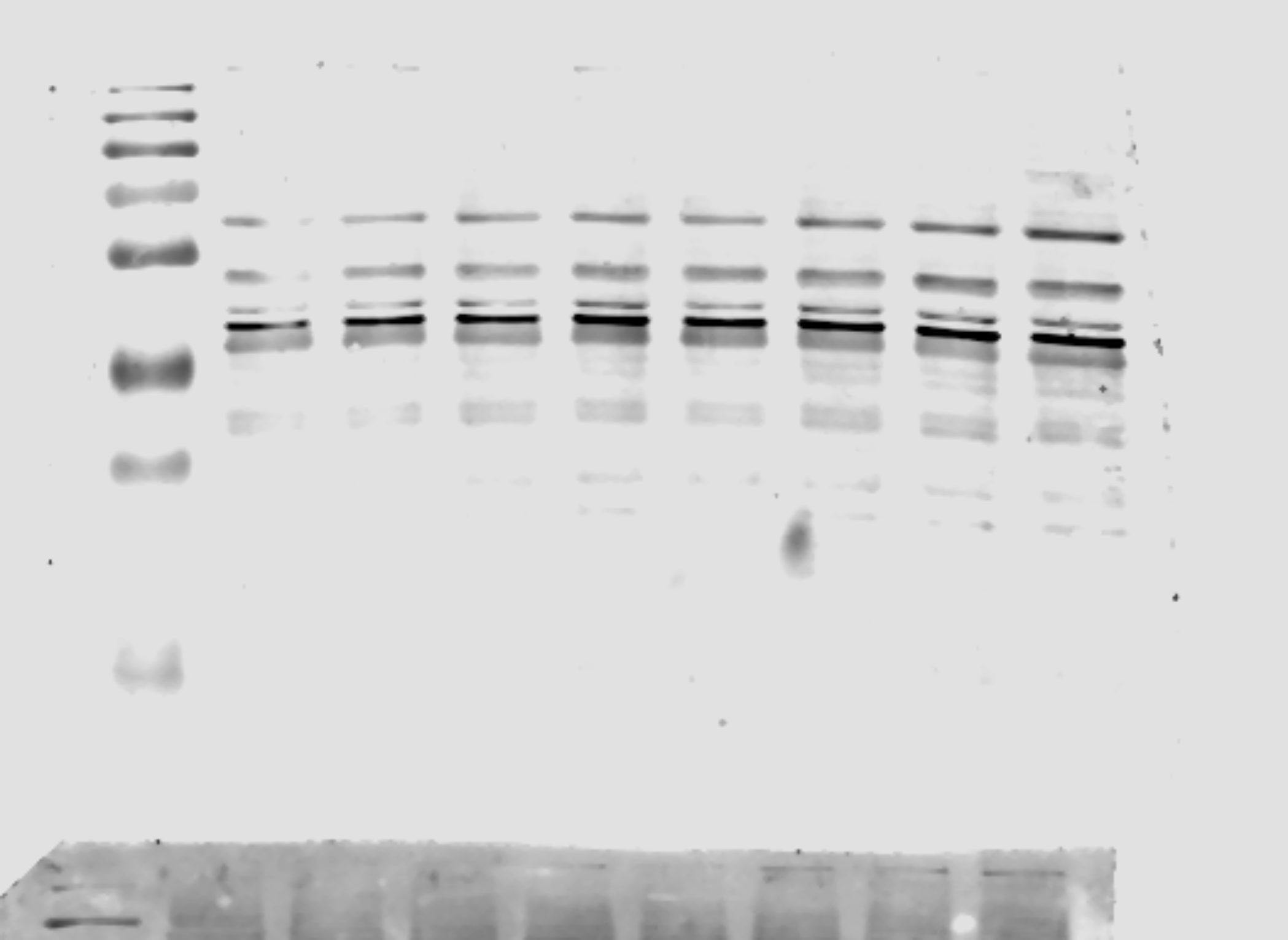

Supplement: Supplementary file 1 — Additional file 1: Additional Fig. 1: Gene expression in neuroblastoma tumors and cell lines. The figure includes correlation study and survival analysis from NB patient datasets and flow cytometry analysis from neuroblastoma cell lines. Additional Fig. 2: Validation of in vitro hypoxia cytometry. Additional Fig. 3: Effect of human recombinant MIF and siCXCR4 in neuroblastoma cell lines. Additional Fig. 4: Membrane CD74 levels by flow cytometry. Additional Fig. 5: Flow cytometry density plots of 4-IPP activity. Additional Fig. 6: LAN-1 viability exposed to CM-NB, CM-BM and treated with AMD-3100 and 4-IPP. LAN-1 response to chemotherapeutic agents when exposed to CM-CNT and treated with 4-IPP. Additional Table 1: Bone marrow samples. Additional Table 2: Primer list, and Additional Table 3: Antibody list. [file 12885_2022_9725_MOESM1_ESM.zip › blot 10.tif]

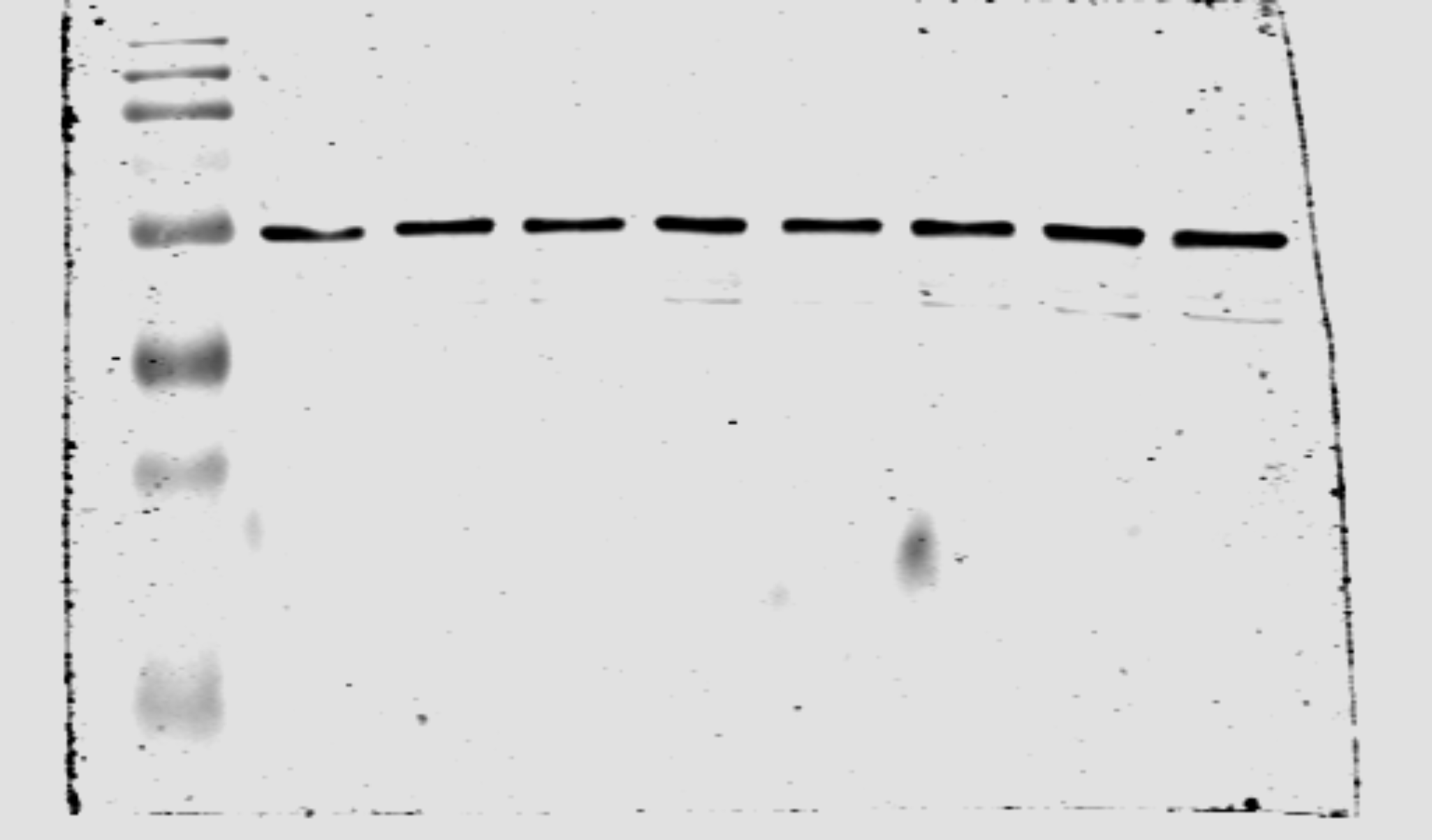

Supplement: Supplementary file 1 — Additional file 1: Additional Fig. 1: Gene expression in neuroblastoma tumors and cell lines. The figure includes correlation study and survival analysis from NB patient datasets and flow cytometry analysis from neuroblastoma cell lines. Additional Fig. 2: Validation of in vitro hypoxia cytometry. Additional Fig. 3: Effect of human recombinant MIF and siCXCR4 in neuroblastoma cell lines. Additional Fig. 4: Membrane CD74 levels by flow cytometry. Additional Fig. 5: Flow cytometry density plots of 4-IPP activity. Additional Fig. 6: LAN-1 viability exposed to CM-NB, CM-BM and treated with AMD-3100 and 4-IPP. LAN-1 response to chemotherapeutic agents when exposed to CM-CNT and treated with 4-IPP. Additional Table 1: Bone marrow samples. Additional Table 2: Primer list, and Additional Table 3: Antibody list. [file 12885_2022_9725_MOESM1_ESM.zip › blot 11.tif]

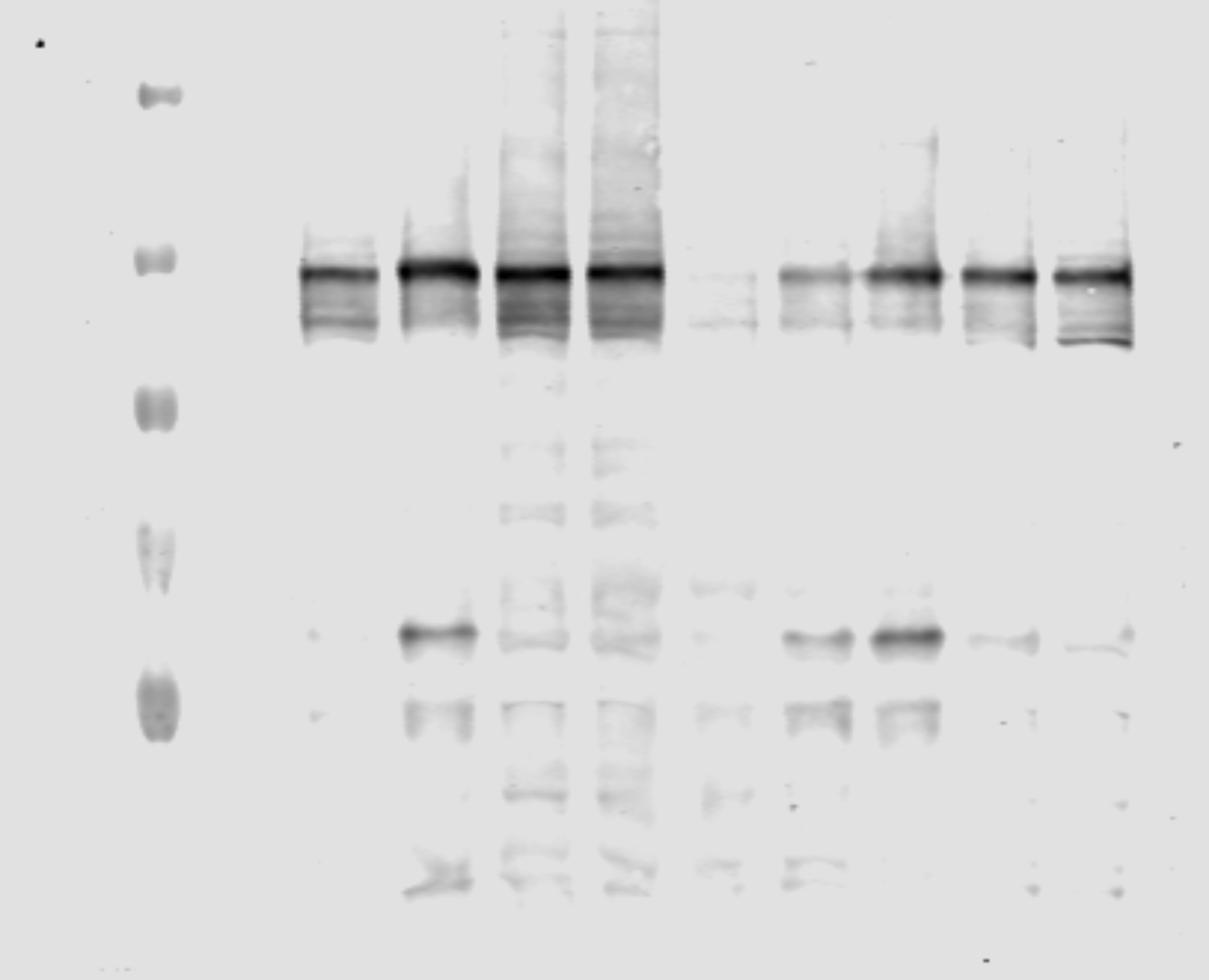

Supplement: Supplementary file 1 — Additional file 1: Additional Fig. 1: Gene expression in neuroblastoma tumors and cell lines. The figure includes correlation study and survival analysis from NB patient datasets and flow cytometry analysis from neuroblastoma cell lines. Additional Fig. 2: Validation of in vitro hypoxia cytometry. Additional Fig. 3: Effect of human recombinant MIF and siCXCR4 in neuroblastoma cell lines. Additional Fig. 4: Membrane CD74 levels by flow cytometry. Additional Fig. 5: Flow cytometry density plots of 4-IPP activity. Additional Fig. 6: LAN-1 viability exposed to CM-NB, CM-BM and treated with AMD-3100 and 4-IPP. LAN-1 response to chemotherapeutic agents when exposed to CM-CNT and treated with 4-IPP. Additional Table 1: Bone marrow samples. Additional Table 2: Primer list, and Additional Table 3: Antibody list. [file 12885_2022_9725_MOESM1_ESM.zip › blot 12.tif]

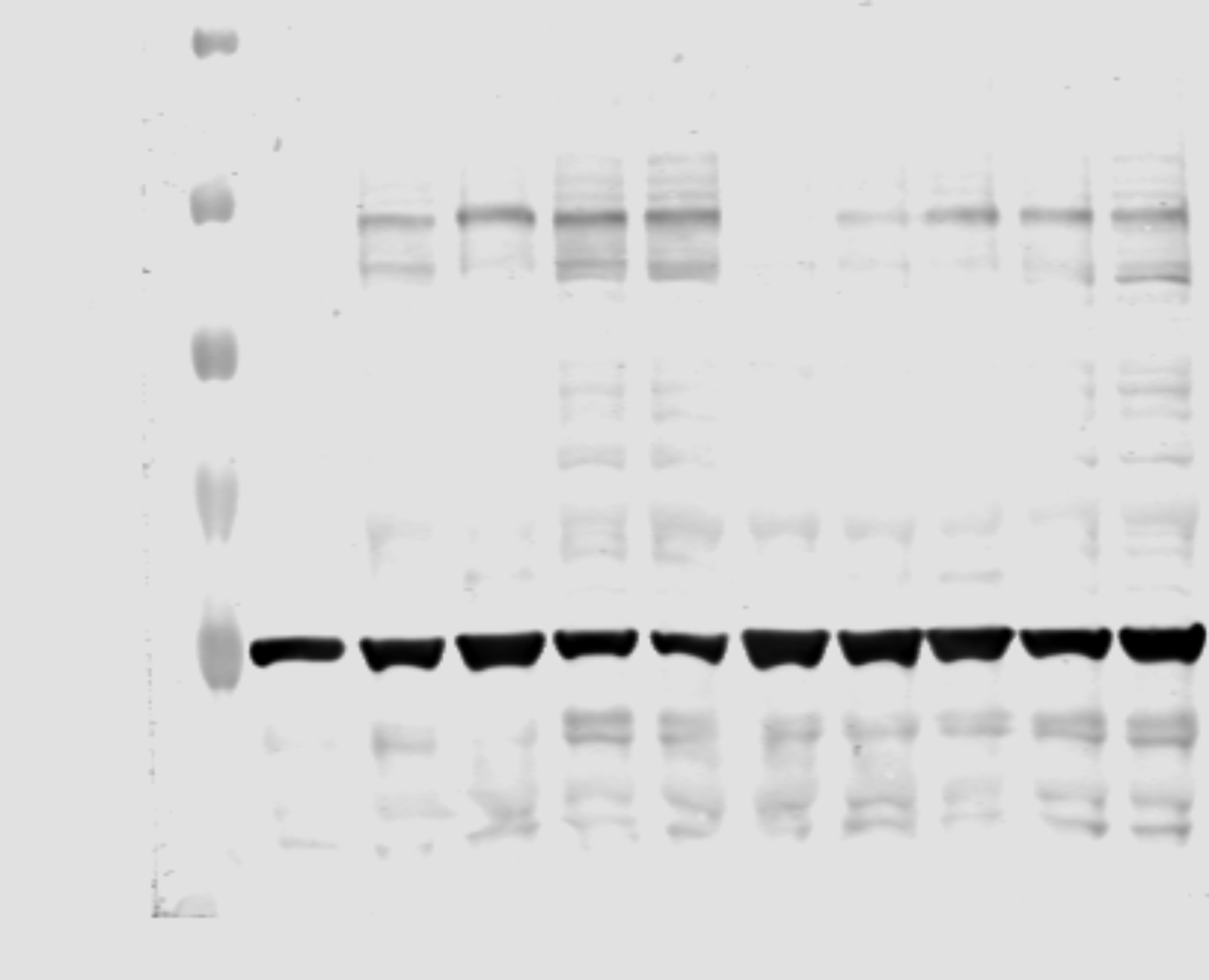

Supplement: Supplementary file 1 — Additional file 1: Additional Fig. 1: Gene expression in neuroblastoma tumors and cell lines. The figure includes correlation study and survival analysis from NB patient datasets and flow cytometry analysis from neuroblastoma cell lines. Additional Fig. 2: Validation of in vitro hypoxia cytometry. Additional Fig. 3: Effect of human recombinant MIF and siCXCR4 in neuroblastoma cell lines. Additional Fig. 4: Membrane CD74 levels by flow cytometry. Additional Fig. 5: Flow cytometry density plots of 4-IPP activity. Additional Fig. 6: LAN-1 viability exposed to CM-NB, CM-BM and treated with AMD-3100 and 4-IPP. LAN-1 response to chemotherapeutic agents when exposed to CM-CNT and treated with 4-IPP. Additional Table 1: Bone marrow samples. Additional Table 2: Primer list, and Additional Table 3: Antibody list. [file 12885_2022_9725_MOESM1_ESM.zip › blot 13.tif]

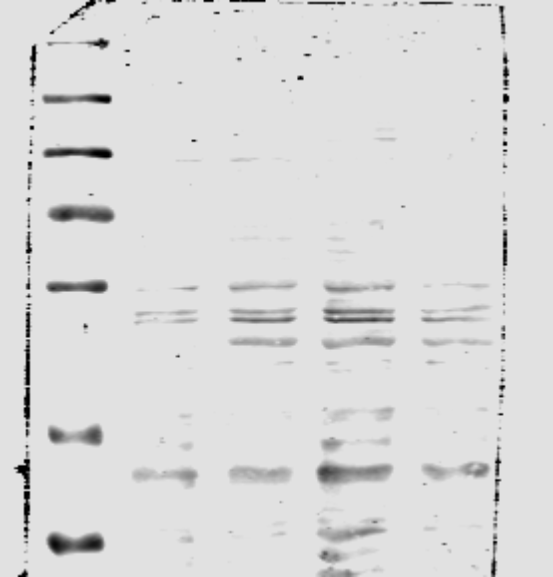

Supplement: Supplementary file 1 — Additional file 1: Additional Fig. 1: Gene expression in neuroblastoma tumors and cell lines. The figure includes correlation study and survival analysis from NB patient datasets and flow cytometry analysis from neuroblastoma cell lines. Additional Fig. 2: Validation of in vitro hypoxia cytometry. Additional Fig. 3: Effect of human recombinant MIF and siCXCR4 in neuroblastoma cell lines. Additional Fig. 4: Membrane CD74 levels by flow cytometry. Additional Fig. 5: Flow cytometry density plots of 4-IPP activity. Additional Fig. 6: LAN-1 viability exposed to CM-NB, CM-BM and treated with AMD-3100 and 4-IPP. LAN-1 response to chemotherapeutic agents when exposed to CM-CNT and treated with 4-IPP. Additional Table 1: Bone marrow samples. Additional Table 2: Primer list, and Additional Table 3: Antibody list. [file 12885_2022_9725_MOESM1_ESM.zip › blot 14.tif]

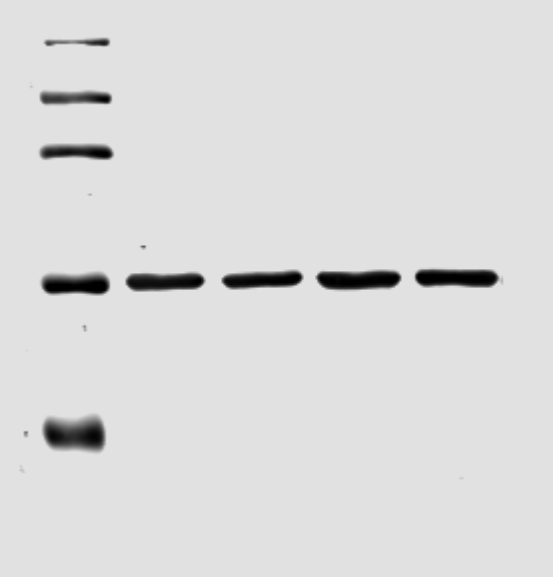

Supplement: Supplementary file 1 — Additional file 1: Additional Fig. 1: Gene expression in neuroblastoma tumors and cell lines. The figure includes correlation study and survival analysis from NB patient datasets and flow cytometry analysis from neuroblastoma cell lines. Additional Fig. 2: Validation of in vitro hypoxia cytometry. Additional Fig. 3: Effect of human recombinant MIF and siCXCR4 in neuroblastoma cell lines. Additional Fig. 4: Membrane CD74 levels by flow cytometry. Additional Fig. 5: Flow cytometry density plots of 4-IPP activity. Additional Fig. 6: LAN-1 viability exposed to CM-NB, CM-BM and treated with AMD-3100 and 4-IPP. LAN-1 response to chemotherapeutic agents when exposed to CM-CNT and treated with 4-IPP. Additional Table 1: Bone marrow samples. Additional Table 2: Primer list, and Additional Table 3: Antibody list. [file 12885_2022_9725_MOESM1_ESM.zip › blot 15.tif]

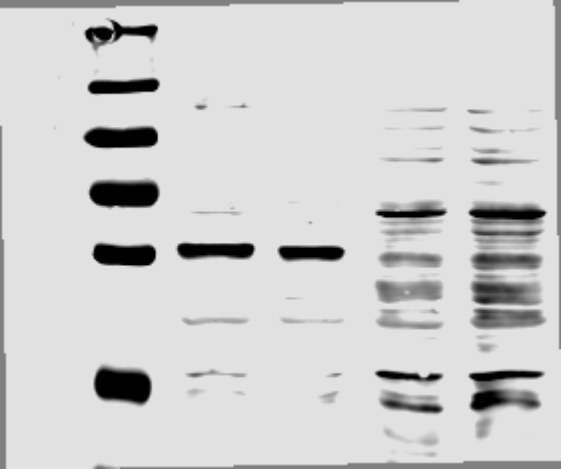

Supplement: Supplementary file 1 — Additional file 1: Additional Fig. 1: Gene expression in neuroblastoma tumors and cell lines. The figure includes correlation study and survival analysis from NB patient datasets and flow cytometry analysis from neuroblastoma cell lines. Additional Fig. 2: Validation of in vitro hypoxia cytometry. Additional Fig. 3: Effect of human recombinant MIF and siCXCR4 in neuroblastoma cell lines. Additional Fig. 4: Membrane CD74 levels by flow cytometry. Additional Fig. 5: Flow cytometry density plots of 4-IPP activity. Additional Fig. 6: LAN-1 viability exposed to CM-NB, CM-BM and treated with AMD-3100 and 4-IPP. LAN-1 response to chemotherapeutic agents when exposed to CM-CNT and treated with 4-IPP. Additional Table 1: Bone marrow samples. Additional Table 2: Primer list, and Additional Table 3: Antibody list. [file 12885_2022_9725_MOESM1_ESM.zip › blot 16.tif]

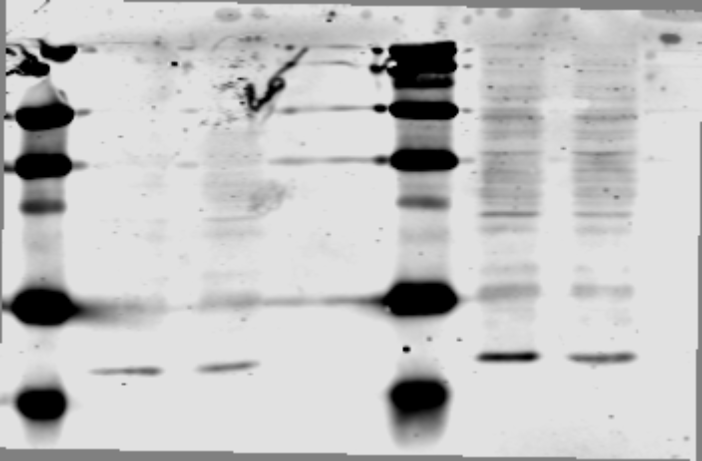

Supplement: Supplementary file 1 — Additional file 1: Additional Fig. 1: Gene expression in neuroblastoma tumors and cell lines. The figure includes correlation study and survival analysis from NB patient datasets and flow cytometry analysis from neuroblastoma cell lines. Additional Fig. 2: Validation of in vitro hypoxia cytometry. Additional Fig. 3: Effect of human recombinant MIF and siCXCR4 in neuroblastoma cell lines. Additional Fig. 4: Membrane CD74 levels by flow cytometry. Additional Fig. 5: Flow cytometry density plots of 4-IPP activity. Additional Fig. 6: LAN-1 viability exposed to CM-NB, CM-BM and treated with AMD-3100 and 4-IPP. LAN-1 response to chemotherapeutic agents when exposed to CM-CNT and treated with 4-IPP. Additional Table 1: Bone marrow samples. Additional Table 2: Primer list, and Additional Table 3: Antibody list. [file 12885_2022_9725_MOESM1_ESM.zip › blot 17.tif]

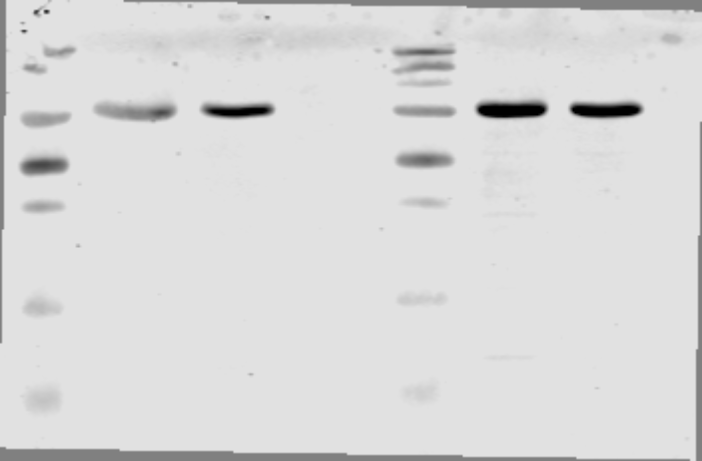

Supplement: Supplementary file 1 — Additional file 1: Additional Fig. 1: Gene expression in neuroblastoma tumors and cell lines. The figure includes correlation study and survival analysis from NB patient datasets and flow cytometry analysis from neuroblastoma cell lines. Additional Fig. 2: Validation of in vitro hypoxia cytometry. Additional Fig. 3: Effect of human recombinant MIF and siCXCR4 in neuroblastoma cell lines. Additional Fig. 4: Membrane CD74 levels by flow cytometry. Additional Fig. 5: Flow cytometry density plots of 4-IPP activity. Additional Fig. 6: LAN-1 viability exposed to CM-NB, CM-BM and treated with AMD-3100 and 4-IPP. LAN-1 response to chemotherapeutic agents when exposed to CM-CNT and treated with 4-IPP. Additional Table 1: Bone marrow samples. Additional Table 2: Primer list, and Additional Table 3: Antibody list. [file 12885_2022_9725_MOESM1_ESM.zip › blot 18.tif]

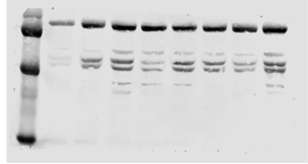

Supplement: Supplementary file 1 — Additional file 1: Additional Fig. 1: Gene expression in neuroblastoma tumors and cell lines. The figure includes correlation study and survival analysis from NB patient datasets and flow cytometry analysis from neuroblastoma cell lines. Additional Fig. 2: Validation of in vitro hypoxia cytometry. Additional Fig. 3: Effect of human recombinant MIF and siCXCR4 in neuroblastoma cell lines. Additional Fig. 4: Membrane CD74 levels by flow cytometry. Additional Fig. 5: Flow cytometry density plots of 4-IPP activity. Additional Fig. 6: LAN-1 viability exposed to CM-NB, CM-BM and treated with AMD-3100 and 4-IPP. LAN-1 response to chemotherapeutic agents when exposed to CM-CNT and treated with 4-IPP. Additional Table 1: Bone marrow samples. Additional Table 2: Primer list, and Additional Table 3: Antibody list. [file 12885_2022_9725_MOESM1_ESM.zip › blot 2.png]

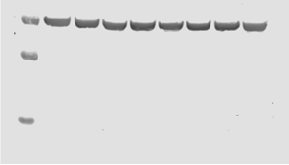

Supplement: Supplementary file 1 — Additional file 1: Additional Fig. 1: Gene expression in neuroblastoma tumors and cell lines. The figure includes correlation study and survival analysis from NB patient datasets and flow cytometry analysis from neuroblastoma cell lines. Additional Fig. 2: Validation of in vitro hypoxia cytometry. Additional Fig. 3: Effect of human recombinant MIF and siCXCR4 in neuroblastoma cell lines. Additional Fig. 4: Membrane CD74 levels by flow cytometry. Additional Fig. 5: Flow cytometry density plots of 4-IPP activity. Additional Fig. 6: LAN-1 viability exposed to CM-NB, CM-BM and treated with AMD-3100 and 4-IPP. LAN-1 response to chemotherapeutic agents when exposed to CM-CNT and treated with 4-IPP. Additional Table 1: Bone marrow samples. Additional Table 2: Primer list, and Additional Table 3: Antibody list. [file 12885_2022_9725_MOESM1_ESM.zip › blot 3.tif]

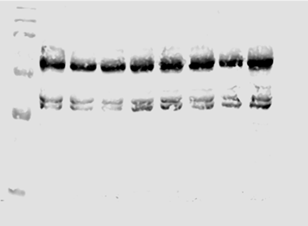

Supplement: Supplementary file 1 — Additional file 1: Additional Fig. 1: Gene expression in neuroblastoma tumors and cell lines. The figure includes correlation study and survival analysis from NB patient datasets and flow cytometry analysis from neuroblastoma cell lines. Additional Fig. 2: Validation of in vitro hypoxia cytometry. Additional Fig. 3: Effect of human recombinant MIF and siCXCR4 in neuroblastoma cell lines. Additional Fig. 4: Membrane CD74 levels by flow cytometry. Additional Fig. 5: Flow cytometry density plots of 4-IPP activity. Additional Fig. 6: LAN-1 viability exposed to CM-NB, CM-BM and treated with AMD-3100 and 4-IPP. LAN-1 response to chemotherapeutic agents when exposed to CM-CNT and treated with 4-IPP. Additional Table 1: Bone marrow samples. Additional Table 2: Primer list, and Additional Table 3: Antibody list. [file 12885_2022_9725_MOESM1_ESM.zip › blot 4.tif]

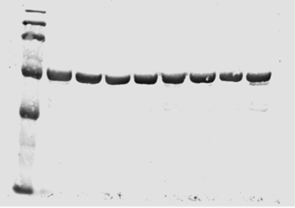

Supplement: Supplementary file 1 — Additional file 1: Additional Fig. 1: Gene expression in neuroblastoma tumors and cell lines. The figure includes correlation study and survival analysis from NB patient datasets and flow cytometry analysis from neuroblastoma cell lines. Additional Fig. 2: Validation of in vitro hypoxia cytometry. Additional Fig. 3: Effect of human recombinant MIF and siCXCR4 in neuroblastoma cell lines. Additional Fig. 4: Membrane CD74 levels by flow cytometry. Additional Fig. 5: Flow cytometry density plots of 4-IPP activity. Additional Fig. 6: LAN-1 viability exposed to CM-NB, CM-BM and treated with AMD-3100 and 4-IPP. LAN-1 response to chemotherapeutic agents when exposed to CM-CNT and treated with 4-IPP. Additional Table 1: Bone marrow samples. Additional Table 2: Primer list, and Additional Table 3: Antibody list. [file 12885_2022_9725_MOESM1_ESM.zip › blot 5.tif]

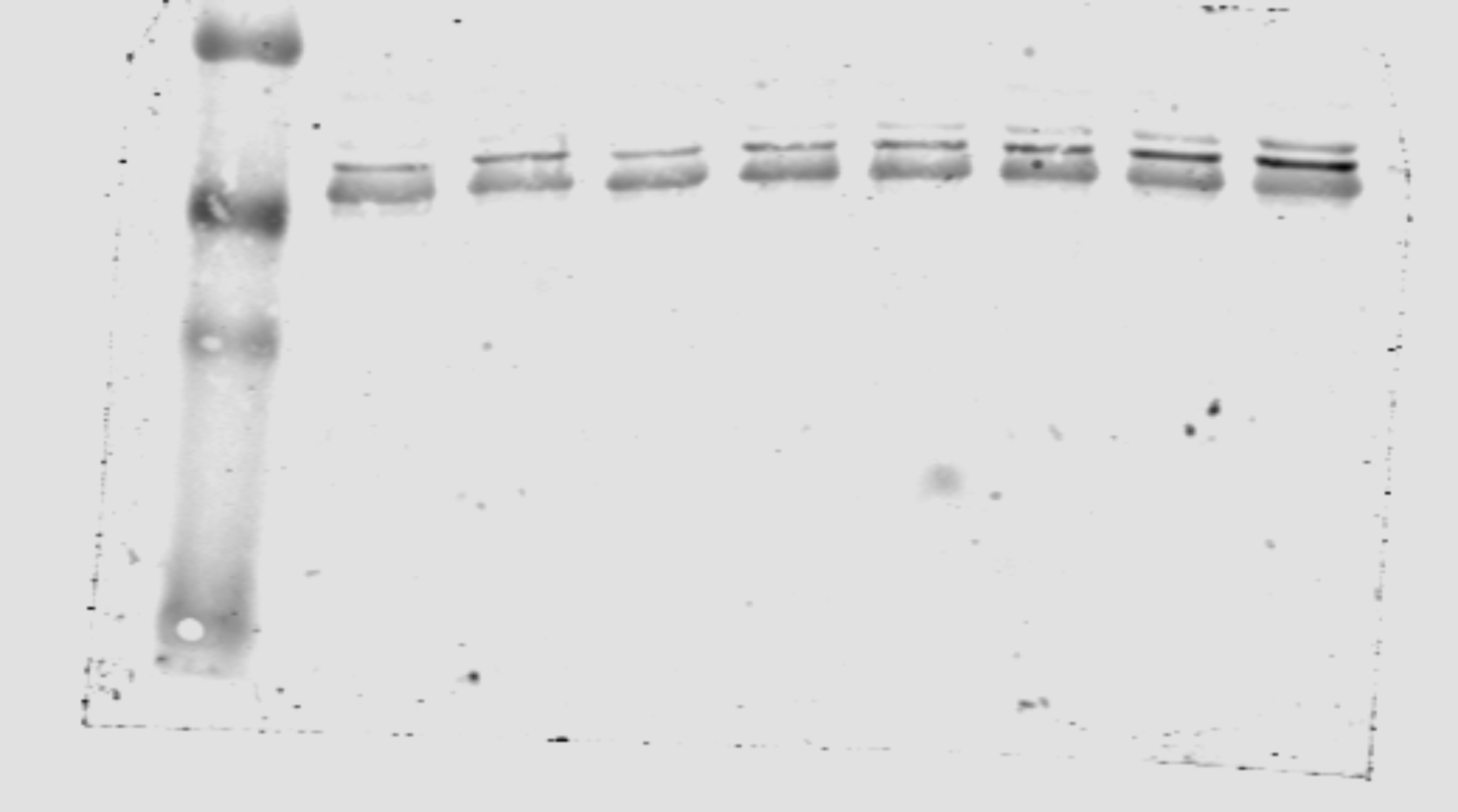

Supplement: Supplementary file 1 — Additional file 1: Additional Fig. 1: Gene expression in neuroblastoma tumors and cell lines. The figure includes correlation study and survival analysis from NB patient datasets and flow cytometry analysis from neuroblastoma cell lines. Additional Fig. 2: Validation of in vitro hypoxia cytometry. Additional Fig. 3: Effect of human recombinant MIF and siCXCR4 in neuroblastoma cell lines. Additional Fig. 4: Membrane CD74 levels by flow cytometry. Additional Fig. 5: Flow cytometry density plots of 4-IPP activity. Additional Fig. 6: LAN-1 viability exposed to CM-NB, CM-BM and treated with AMD-3100 and 4-IPP. LAN-1 response to chemotherapeutic agents when exposed to CM-CNT and treated with 4-IPP. Additional Table 1: Bone marrow samples. Additional Table 2: Primer list, and Additional Table 3: Antibody list. [file 12885_2022_9725_MOESM1_ESM.zip › blot 6.tif]

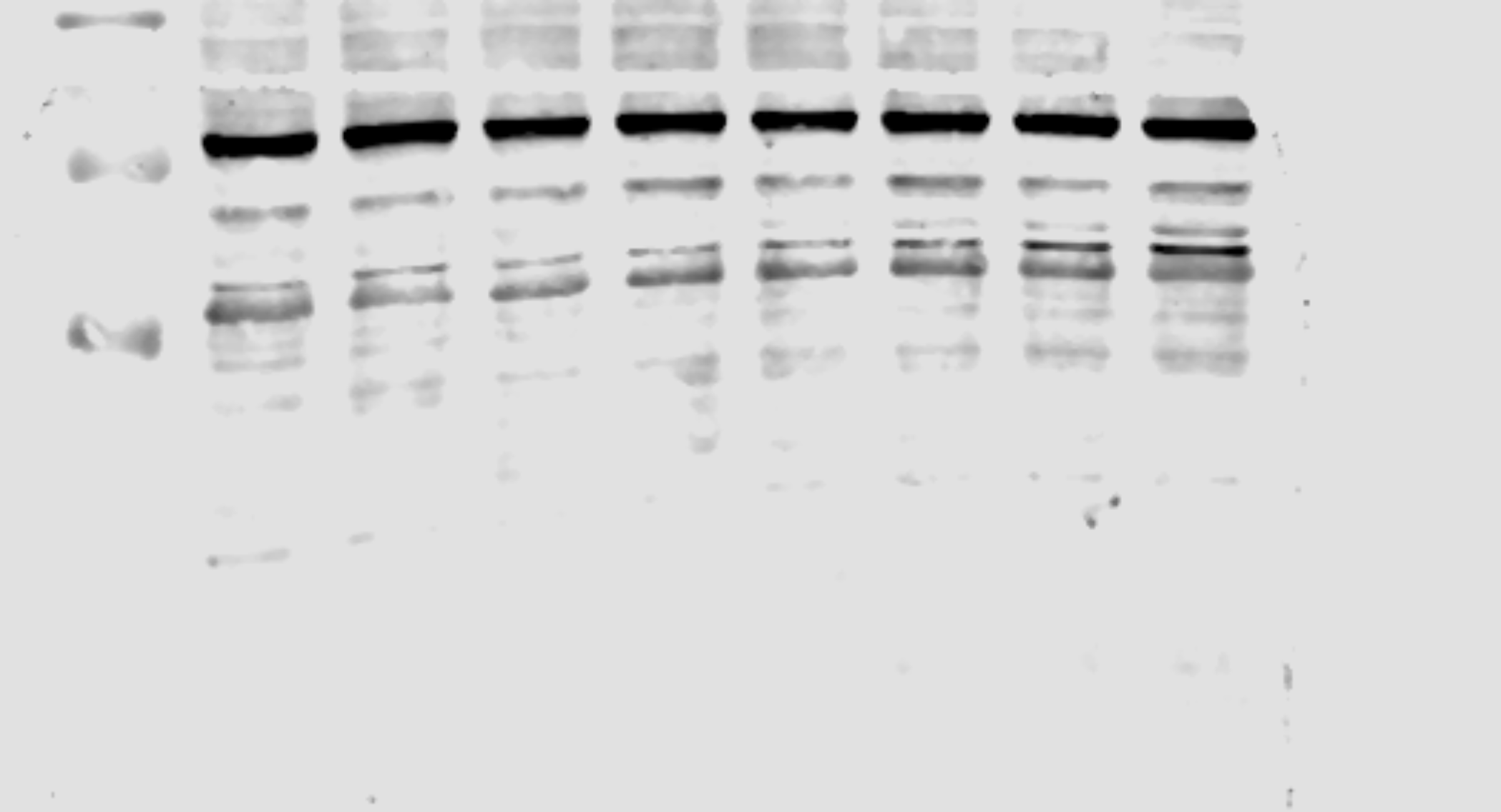

Supplement: Supplementary file 1 — Additional file 1: Additional Fig. 1: Gene expression in neuroblastoma tumors and cell lines. The figure includes correlation study and survival analysis from NB patient datasets and flow cytometry analysis from neuroblastoma cell lines. Additional Fig. 2: Validation of in vitro hypoxia cytometry. Additional Fig. 3: Effect of human recombinant MIF and siCXCR4 in neuroblastoma cell lines. Additional Fig. 4: Membrane CD74 levels by flow cytometry. Additional Fig. 5: Flow cytometry density plots of 4-IPP activity. Additional Fig. 6: LAN-1 viability exposed to CM-NB, CM-BM and treated with AMD-3100 and 4-IPP. LAN-1 response to chemotherapeutic agents when exposed to CM-CNT and treated with 4-IPP. Additional Table 1: Bone marrow samples. Additional Table 2: Primer list, and Additional Table 3: Antibody list. [file 12885_2022_9725_MOESM1_ESM.zip › blot 7.tif]

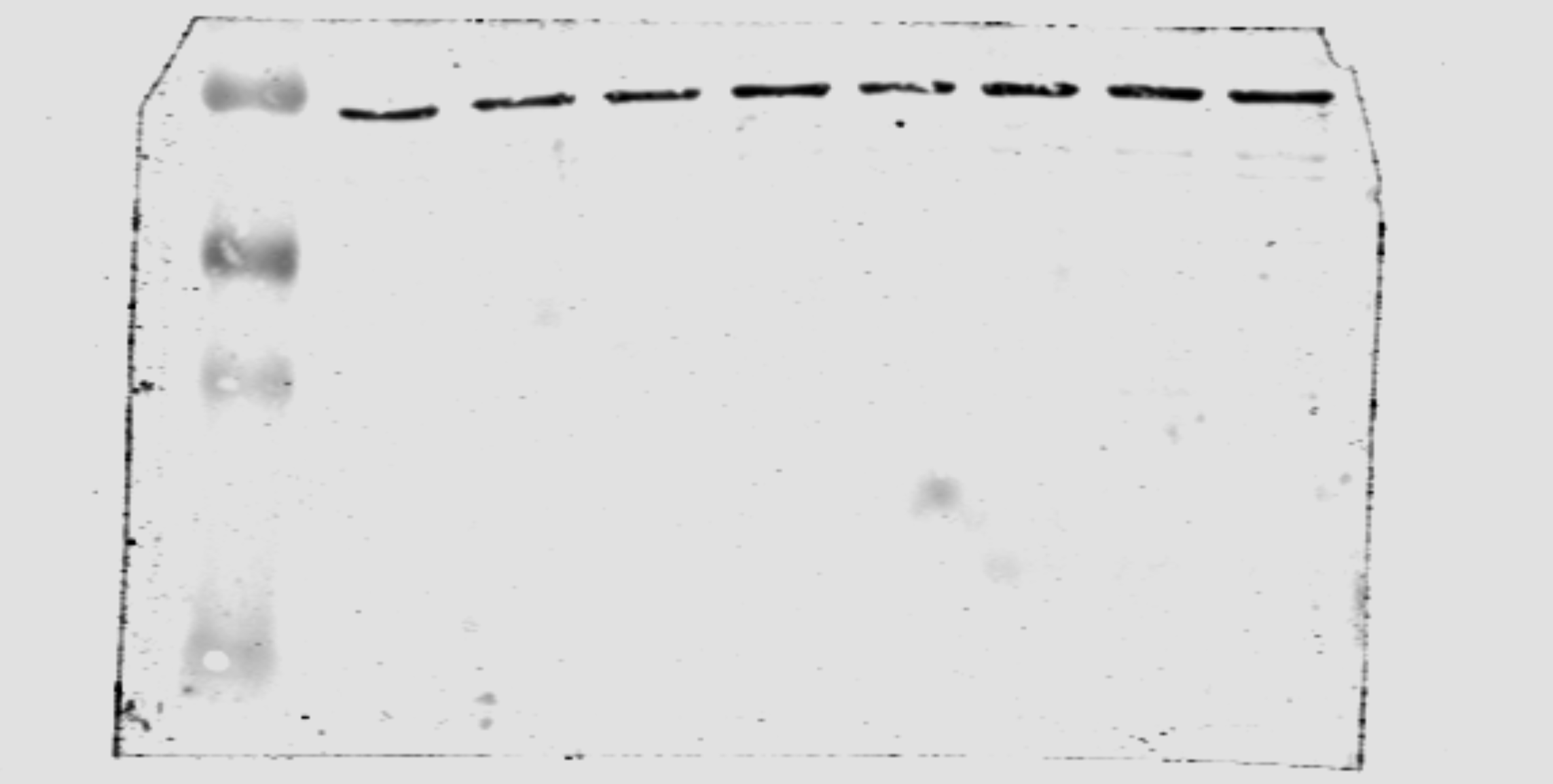

Supplement: Supplementary file 1 — Additional file 1: Additional Fig. 1: Gene expression in neuroblastoma tumors and cell lines. The figure includes correlation study and survival analysis from NB patient datasets and flow cytometry analysis from neuroblastoma cell lines. Additional Fig. 2: Validation of in vitro hypoxia cytometry. Additional Fig. 3: Effect of human recombinant MIF and siCXCR4 in neuroblastoma cell lines. Additional Fig. 4: Membrane CD74 levels by flow cytometry. Additional Fig. 5: Flow cytometry density plots of 4-IPP activity. Additional Fig. 6: LAN-1 viability exposed to CM-NB, CM-BM and treated with AMD-3100 and 4-IPP. LAN-1 response to chemotherapeutic agents when exposed to CM-CNT and treated with 4-IPP. Additional Table 1: Bone marrow samples. Additional Table 2: Primer list, and Additional Table 3: Antibody list. [file 12885_2022_9725_MOESM1_ESM.zip › blot 8.tif]

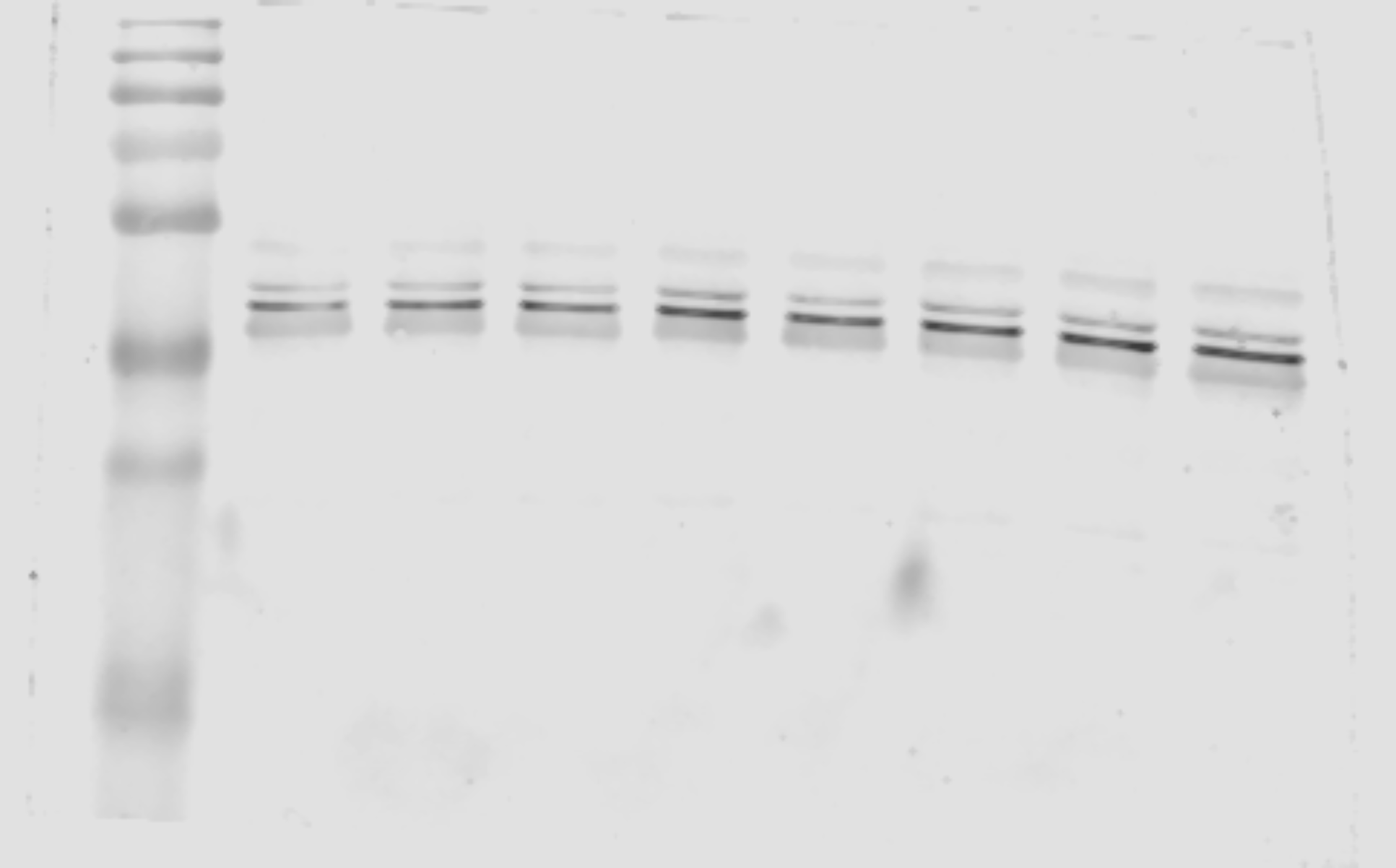

Supplement: Supplementary file 1 — Additional file 1: Additional Fig. 1: Gene expression in neuroblastoma tumors and cell lines. The figure includes correlation study and survival analysis from NB patient datasets and flow cytometry analysis from neuroblastoma cell lines. Additional Fig. 2: Validation of in vitro hypoxia cytometry. Additional Fig. 3: Effect of human recombinant MIF and siCXCR4 in neuroblastoma cell lines. Additional Fig. 4: Membrane CD74 levels by flow cytometry. Additional Fig. 5: Flow cytometry density plots of 4-IPP activity. Additional Fig. 6: LAN-1 viability exposed to CM-NB, CM-BM and treated with AMD-3100 and 4-IPP. LAN-1 response to chemotherapeutic agents when exposed to CM-CNT and treated with 4-IPP. Additional Table 1: Bone marrow samples. Additional Table 2: Primer list, and Additional Table 3: Antibody list. [file 12885_2022_9725_MOESM1_ESM.zip › blot 9.tif]

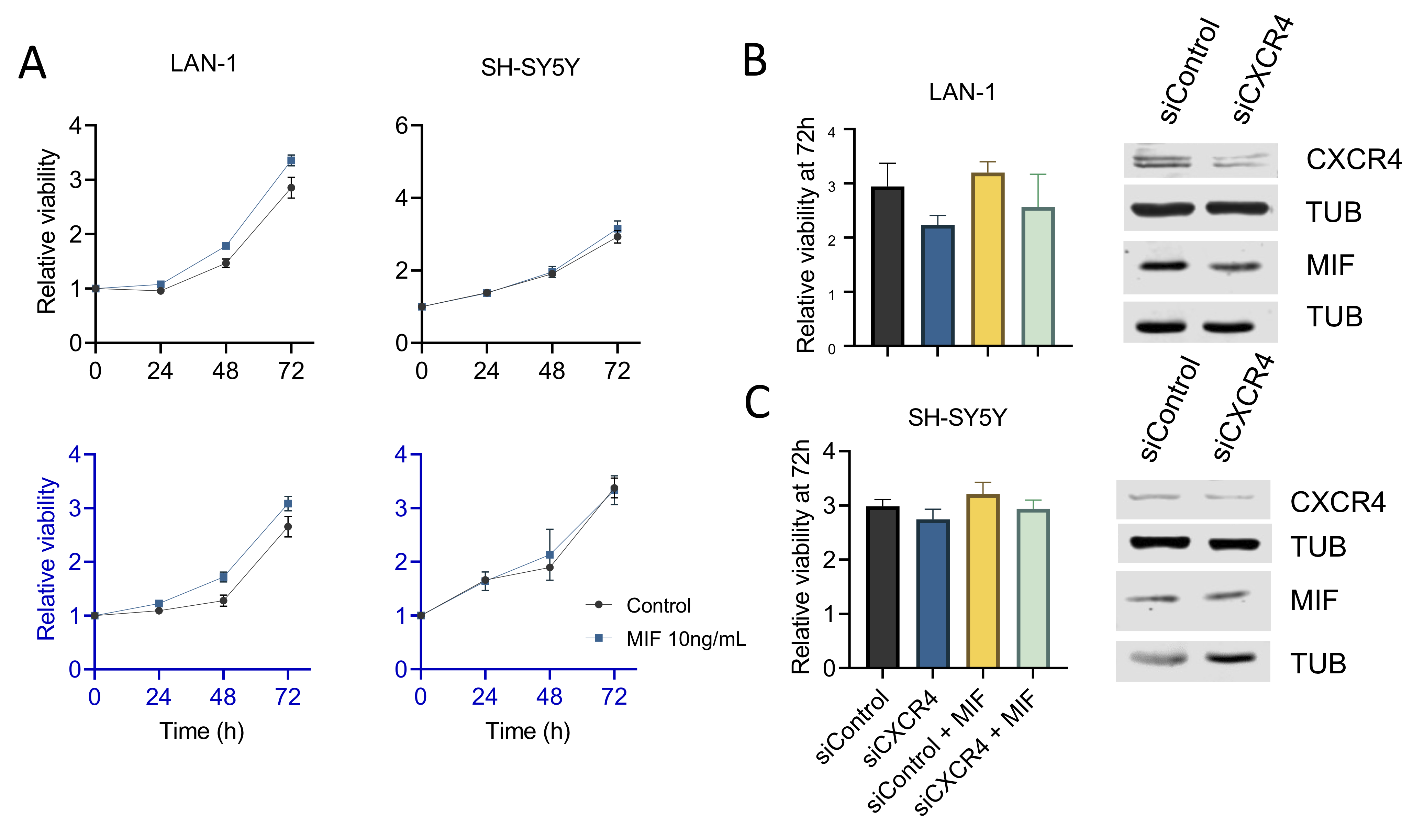

Supplement: Supplementary file 1 — Additional file 1: Additional Fig. 1: Gene expression in neuroblastoma tumors and cell lines. The figure includes correlation study and survival analysis from NB patient datasets and flow cytometry analysis from neuroblastoma cell lines. Additional Fig. 2: Validation of in vitro hypoxia cytometry. Additional Fig. 3: Effect of human recombinant MIF and siCXCR4 in neuroblastoma cell lines. Additional Fig. 4: Membrane CD74 levels by flow cytometry. Additional Fig. 5: Flow cytometry density plots of 4-IPP activity. Additional Fig. 6: LAN-1 viability exposed to CM-NB, CM-BM and treated with AMD-3100 and 4-IPP. LAN-1 response to chemotherapeutic agents when exposed to CM-CNT and treated with 4-IPP. Additional Table 1: Bone marrow samples. Additional Table 2: Primer list, and Additional Table 3: Antibody list. [file 12885_2022_9725_MOESM1_ESM.zip › Figure S3.tiff]

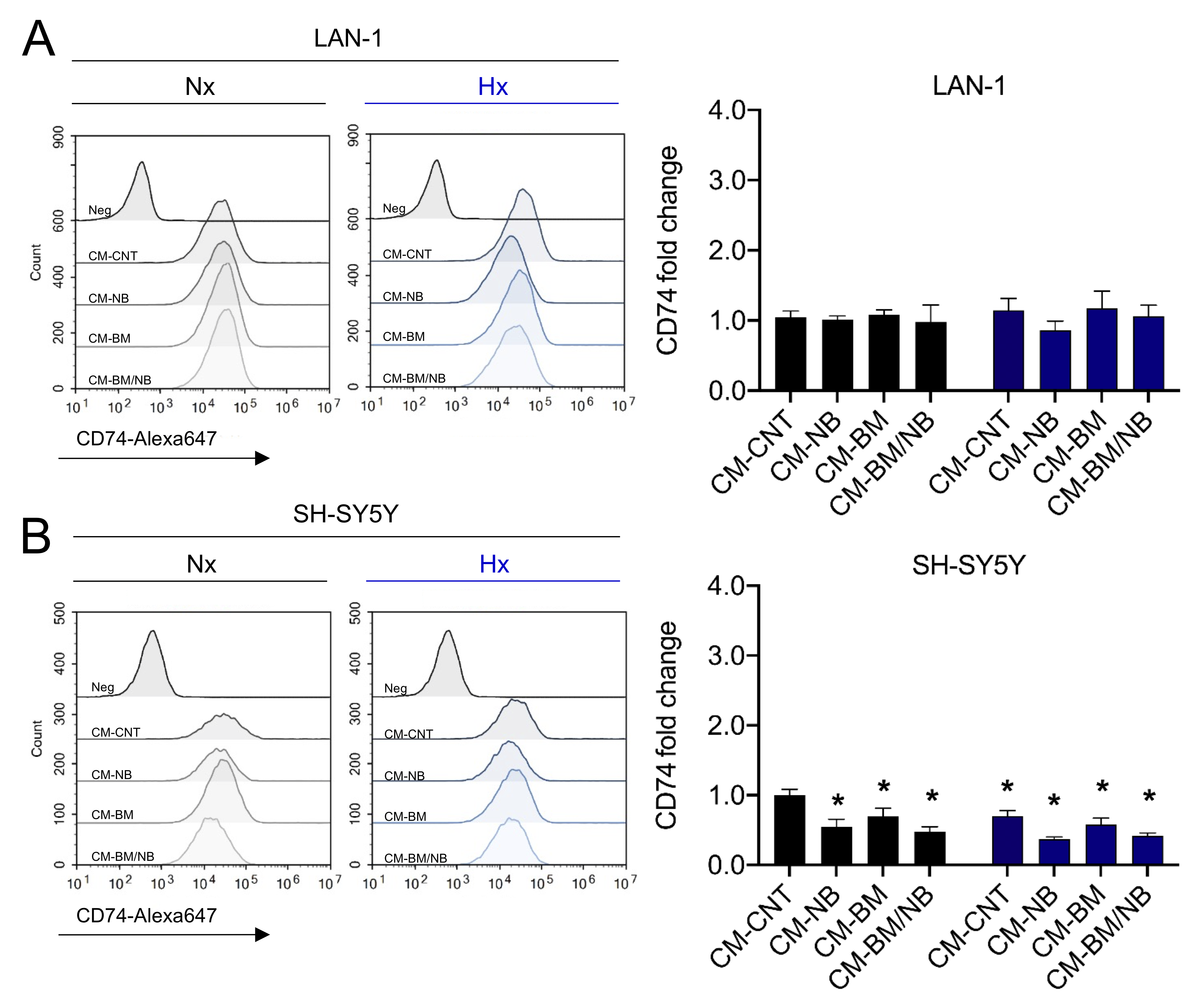

Supplement: Supplementary file 1 — Additional file 1: Additional Fig. 1: Gene expression in neuroblastoma tumors and cell lines. The figure includes correlation study and survival analysis from NB patient datasets and flow cytometry analysis from neuroblastoma cell lines. Additional Fig. 2: Validation of in vitro hypoxia cytometry. Additional Fig. 3: Effect of human recombinant MIF and siCXCR4 in neuroblastoma cell lines. Additional Fig. 4: Membrane CD74 levels by flow cytometry. Additional Fig. 5: Flow cytometry density plots of 4-IPP activity. Additional Fig. 6: LAN-1 viability exposed to CM-NB, CM-BM and treated with AMD-3100 and 4-IPP. LAN-1 response to chemotherapeutic agents when exposed to CM-CNT and treated with 4-IPP. Additional Table 1: Bone marrow samples. Additional Table 2: Primer list, and Additional Table 3: Antibody list. [file 12885_2022_9725_MOESM1_ESM.zip › Figure S4.tiff]

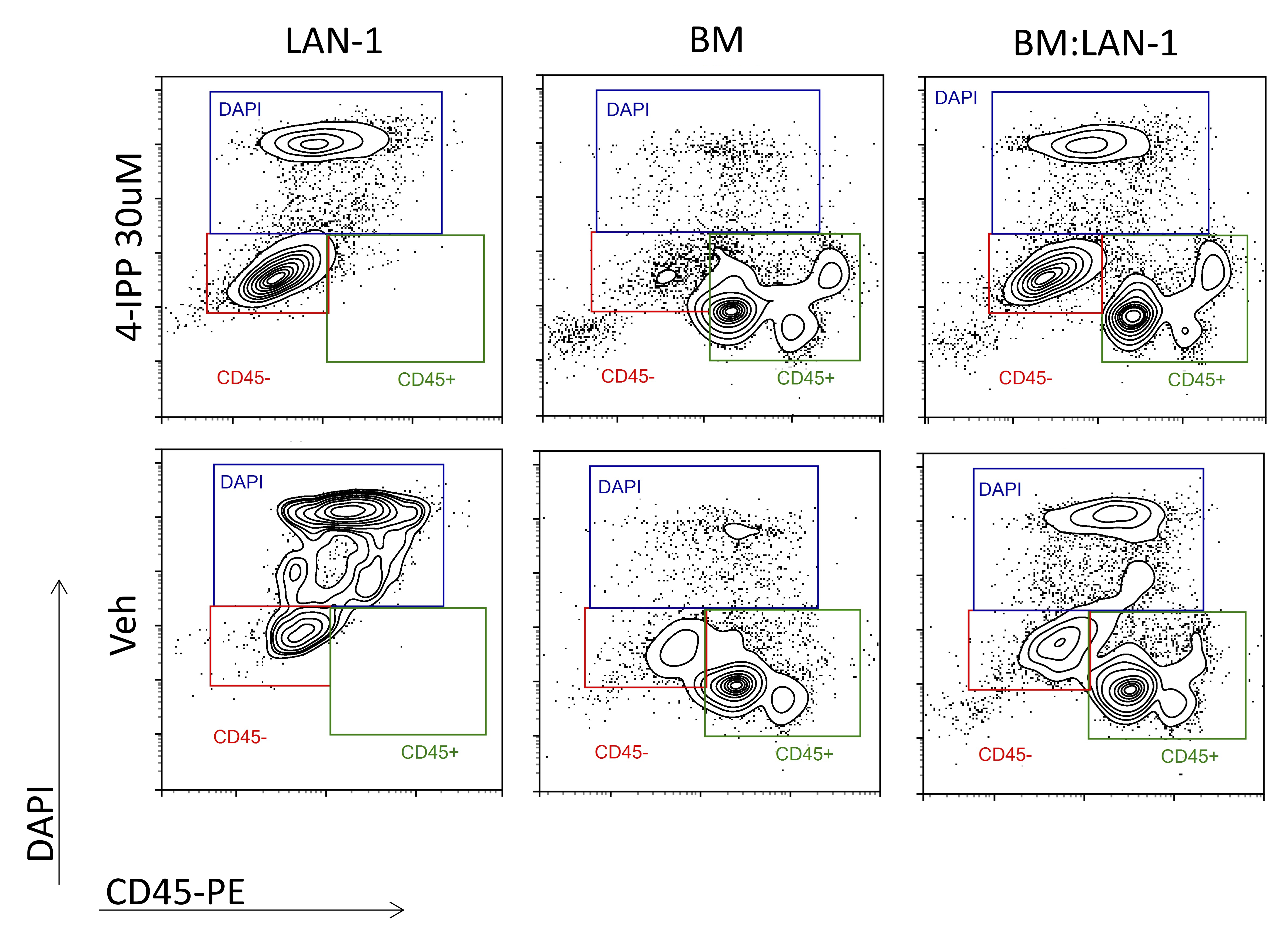

Supplement: Supplementary file 1 — Additional file 1: Additional Fig. 1: Gene expression in neuroblastoma tumors and cell lines. The figure includes correlation study and survival analysis from NB patient datasets and flow cytometry analysis from neuroblastoma cell lines. Additional Fig. 2: Validation of in vitro hypoxia cytometry. Additional Fig. 3: Effect of human recombinant MIF and siCXCR4 in neuroblastoma cell lines. Additional Fig. 4: Membrane CD74 levels by flow cytometry. Additional Fig. 5: Flow cytometry density plots of 4-IPP activity. Additional Fig. 6: LAN-1 viability exposed to CM-NB, CM-BM and treated with AMD-3100 and 4-IPP. LAN-1 response to chemotherapeutic agents when exposed to CM-CNT and treated with 4-IPP. Additional Table 1: Bone marrow samples. Additional Table 2: Primer list, and Additional Table 3: Antibody list. [file 12885_2022_9725_MOESM1_ESM.zip › Figure S5.tiff]

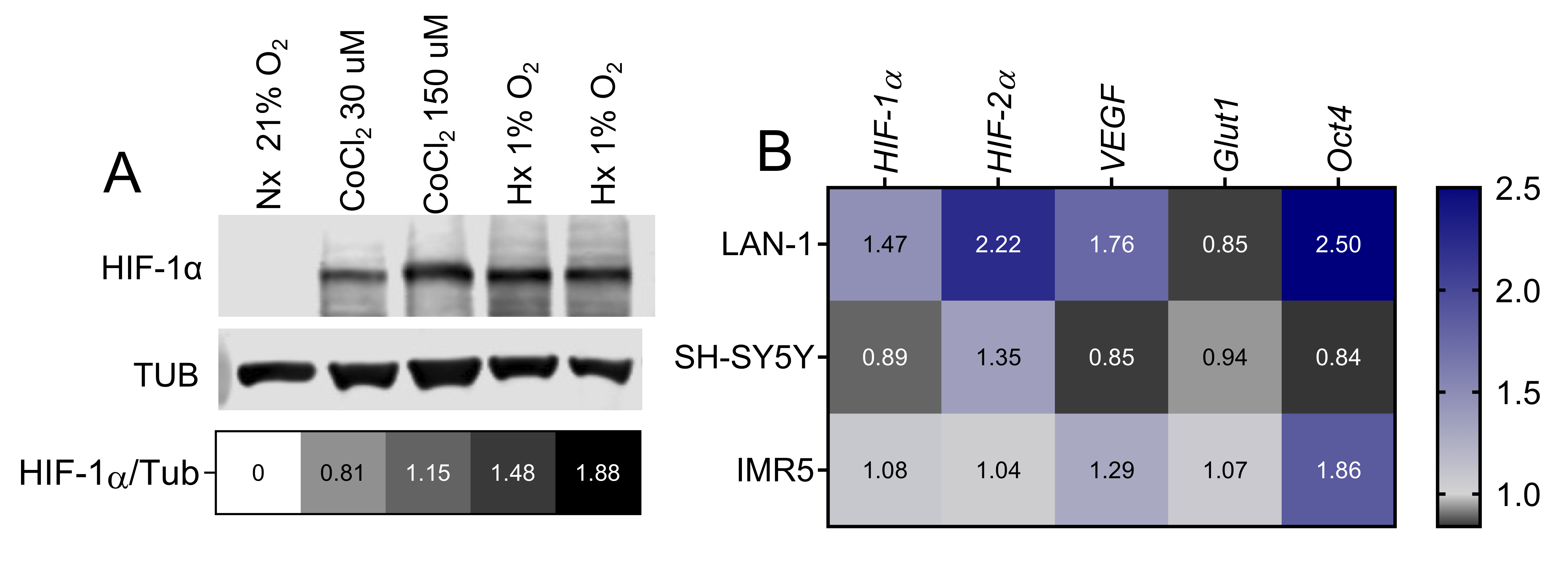

Supplement: Supplementary file 1 — Additional file 1: Additional Fig. 1: Gene expression in neuroblastoma tumors and cell lines. The figure includes correlation study and survival analysis from NB patient datasets and flow cytometry analysis from neuroblastoma cell lines. Additional Fig. 2: Validation of in vitro hypoxia cytometry. Additional Fig. 3: Effect of human recombinant MIF and siCXCR4 in neuroblastoma cell lines. Additional Fig. 4: Membrane CD74 levels by flow cytometry. Additional Fig. 5: Flow cytometry density plots of 4-IPP activity. Additional Fig. 6: LAN-1 viability exposed to CM-NB, CM-BM and treated with AMD-3100 and 4-IPP. LAN-1 response to chemotherapeutic agents when exposed to CM-CNT and treated with 4-IPP. Additional Table 1: Bone marrow samples. Additional Table 2: Primer list, and Additional Table 3: Antibody list. [file 12885_2022_9725_MOESM1_ESM.zip › Figures S2.tiff]

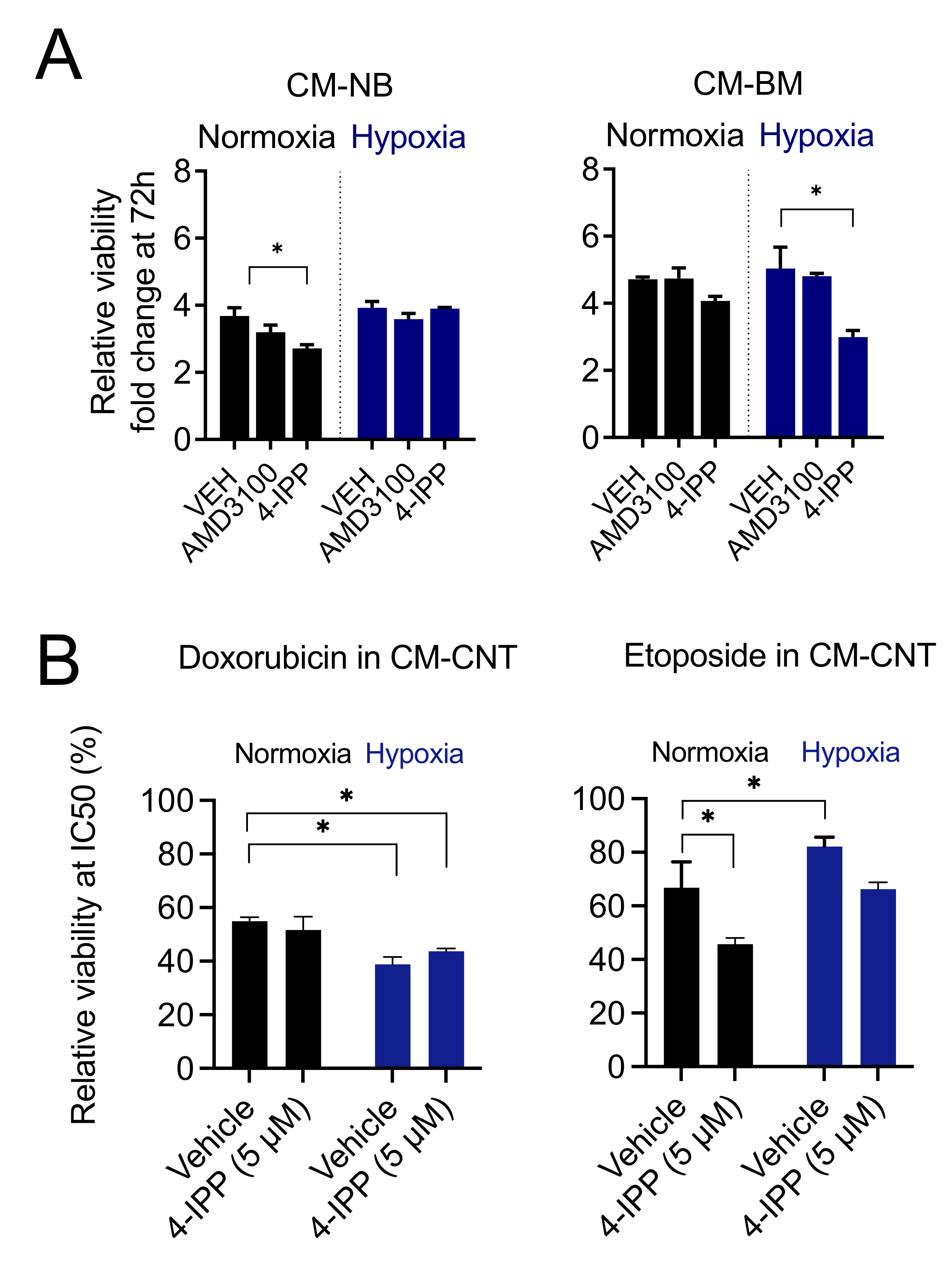

Supplement: Supplementary file 1 — Additional file 1: Additional Fig. 1: Gene expression in neuroblastoma tumors and cell lines. The figure includes correlation study and survival analysis from NB patient datasets and flow cytometry analysis from neuroblastoma cell lines. Additional Fig. 2: Validation of in vitro hypoxia cytometry. Additional Fig. 3: Effect of human recombinant MIF and siCXCR4 in neuroblastoma cell lines. Additional Fig. 4: Membrane CD74 levels by flow cytometry. Additional Fig. 5: Flow cytometry density plots of 4-IPP activity. Additional Fig. 6: LAN-1 viability exposed to CM-NB, CM-BM and treated with AMD-3100 and 4-IPP. LAN-1 response to chemotherapeutic agents when exposed to CM-CNT and treated with 4-IPP. Additional Table 1: Bone marrow samples. Additional Table 2: Primer list, and Additional Table 3: Antibody list. [file 12885_2022_9725_MOESM1_ESM.zip › Figures S6.tiff]
